# Fecal microbiota of the synanthropic golden jackal (*Canis aureus*)

**DOI:** 10.1186/s42523-023-00259-3

**Published:** 2023-08-05

**Authors:** Roi Lapid, Yair Motro, Hillary Craddock, Boris Khalfin, Roni King, Gila Kahila Bar-Gal, Jacob Moran-Gilad

**Affiliations:** 1 The Robert H. Smith Faculty of Agriculture, Food and Environment, The Hebrew University of Jerusalem, P.O.B. 12, 7610001 Rehovot, Israel; 2 Department of Health Policy and Management, School of Public Health, Faculty of Health Sciences, Ben-Gurion University of the Negev, 8410501 Beer-Sheva, Israel; 3 Science and Conservation Division, Israel Nature and Parks Authority, 3 Am Ve’Olamo St., 95463 Jerusalem, Israel

**Keywords:** Fecal microbiota, Microbiome, 16S rRNA amplicon sequencing, Golden jackal (*Canis aureus*), Zoonotic diseases, One-health, Israel, Wildlife

## Abstract

The golden jackal (*Canis aureus*), is a medium canid carnivore widespread throughout the Mediterranean region and expanding into Europe. This species thrives near human settlements and is implicated in zoonoses such as rabies. This study explores for the first time, the golden jackal fecal microbiota. We analyzed 111 fecal samples of wild golden jackals using 16S rRNA amplicon sequencing the connection of the microbiome to animal characteristics, burden of pathogens and geographic and climate characteristics. We further compared the fecal microbiota of the golden jackal to the black-backed jackal and domestic dog. We found that the golden jackal fecal microbiota is dominated by the phyla Bacteroidota, Fusobacteriota and Firmicutes. The golden jackal fecal microbiota was associated with different variables, including geographic region, age-class, exposure to rabies oral vaccine, fecal parasites and toxoplasmosis. A remarkable variation in the relative abundance of different taxa was also found associated with different variables, such as age-class. Linear discriminant analysis effect size (LEfSe) analysis found abundance of specific taxons in each region, Megasphaera genus in group 1, Megamonas genus in group 2 and Bacteroides coprocola species in group 3. We also found a different composition between the fecal microbiota of the golden jackal, blacked-backed jackal and the domestic dog. Furthermore, LEfSe analysis found abundance of Fusobacterium and Bacteroides genera in the golden jackal, Clostridia class in blacked-backed jackal and Megamonas genus in domestic dog. The golden jackal fecal microbiota is influenced by multiple factors including host traits and pathogen burden. The characterization of the microbiota of this thriving species may aid in mapping its spread and proximity to human settlements. Moreover, understanding the jackal microbiota could inform the study of potential animal and human health risks and inform control measures.

**Supplementary Information:**

The online version contains supplementary material available at 10.1186/s42523-023-00259-3.

## Background

The golden jackal (*Canis aureus*; GJ) is a common medium carnivore of the Canidae family, widespread throughout Mediterranean region and expanding into Europe [1, 2]. The Israeli GJ population size has increased drastically countrywide during the last decade, following the invasion of new geographical regions, such as the Negev mostly in association with human settlements [3, 4]. This is due to its excellent reproduction rate (3–8 offspring annually) and its adaptability to a varied diet (vegetables and animals), especially from anthropogenic sources [1, 4–6]. Consequently, the GJ became a synanthropes species, a species that benefit from living in close proximity to humans yet remains beyond their control. The high density of the GJ population in Israel, 25 jackals to 10 km^2^ [7], and its association with humans, makes it a potential exposure for many pathogens, especially zoonotic disease agents.

Pathogens harbored by the GJ are notably understudied. The exceptions are the *Neospora caninum,* a pathogen with agricultural and economic effects, as 3.2% individuals were seropositives [8]. Internal parasites like *Echinococcus granulosus* [9] and *Trichenlla spiralis* [10] which are considered major zoonoses.

Rabies virus is one of the main zoonotic pathogens associated with GJ. Rabies has major public health and economic effects from treating exposed human and causing agricultural damage [11]. Since 2000, an oral Rabies vaccination (ORV), using vaccine-filled baits [12], is employed in Israel, based on a bi-annual distribution. This oral vaccination program resulted in a sharp decline in rabies incidence [13, 14], but an unanticipated resurgence of rabies cases in 2017–2018 was mainly found in GJ and this was presumably from transboundary movement of GJ from unvaccinated neighboring areas [15]. The thriving of the GJ in Israel is mitigated by a national predator control program.

Investigation of animal microbiota can assist in surveillance of animal pathogens, even prior to clinical symptoms [16, 17]. The microbiota describes the microbial communities that inhabit the mucosal body surfaces of animals and humans [18]. Microbiota studies are mainly focusing on humans and animal models, but in the recent years the scope has expanded to veterinary medicine [19, 20] and wild animal conservation through captive and free-roaming wildlife [e.g. wild herbivores [21–23], elephants [24, 25], primates [26–29], carnivores [30–32] and canid species [16, 33–36]. The most densely populated part of the mammalian anatomy is the gastrointestinal (GI) tract, where the microbial cells are thought to outnumber host cells [37]. The GI microbiota is known to be influenced by general exposure, diet and host genetics and different lifestyles [16, 26, 32, 38, 39]. In humans and animals, alterations in the GI microbiota are associated with diseases, including metabolic syndrome, diabetes, obesity, inflammatory bowel disease, asthma, cardiovascular disease, immune-mediated conditions, and neurodevelopmental conditions such as autism spectrum disorder [32, 38, 39].

In human and in wild primate populations, the GI microbiota responds to changes in different diets, a result of habitat and seasonal variation [18, 21, 37]. Howler monkeys (*Simia belzebul*) and Red colobus monkeys (*Procolobus gordonorum*) for example has shown a reduced gut microbiota diversity as a result of fragmentation of the forest [40, 41]. In black-backed jackals (*Canis mesomelas*) gut microbiota diversity was found in association to habitat and seasonality [16]. Seasonal variation effect were identified based on the ratio changes between the Firmicutes to Bacteriodata bacterial phyla. The variation in the ratio of bacteria can be reflected as a predictor of physiological constraints similar to the finding of higher ratio in animals and humans with high energy demands such as lactating females [16].

Microbiota characteristics of random wild animals can be used as a surveillance of animal pathogens to light on possible zoonotic and non-zoonotic agencies. Comparison between urban and rural Coyote (*Canis latrans*) populations, a generalist canid, altered microbiome has found abundance of *Streptococcus* and *Enterococcus*, and poorer average body condition in the rural specimens [42]. In mice (*Mus musculus domestica*) infected with the parasite *Toxoplasma gondii*, the gut microbiota was altered between acute to chronic infection of the parasite [43]. A study in the wild European shag (*Phalacrocorax aristotelis*) has demonstrated marked alternations in the gut microbiome between heavy and low burden helminth infestation [44]. Notably, microbiota studies in wildlife mammal studies are usually based on fecal samples that are prone to the risk of contamination or altered microbial composition due to the time elapsed between sample deposition and collection [45]*.*

The overall knowledge of the jackal microbiota is very limited [16] and there is little if no data on the GJ in Israel although it is an emerging synanthropes species in the human environment. Moreover, the association between the GJ microbiota and features such as the animal characteristics, geographic distribution and burden of pathogens has not been previously explored. Such data are expected to improve our understanding of the GJ as an invading species and inform prevention and control efforts. Hence, the main goal of this study was to characterize the GJ as an emerging synanthropes species in the human environment in Israel, which also account for zoonotic diseases, especially rabies.

## Methods

### Sampling and data collection

**Fig. 1:**
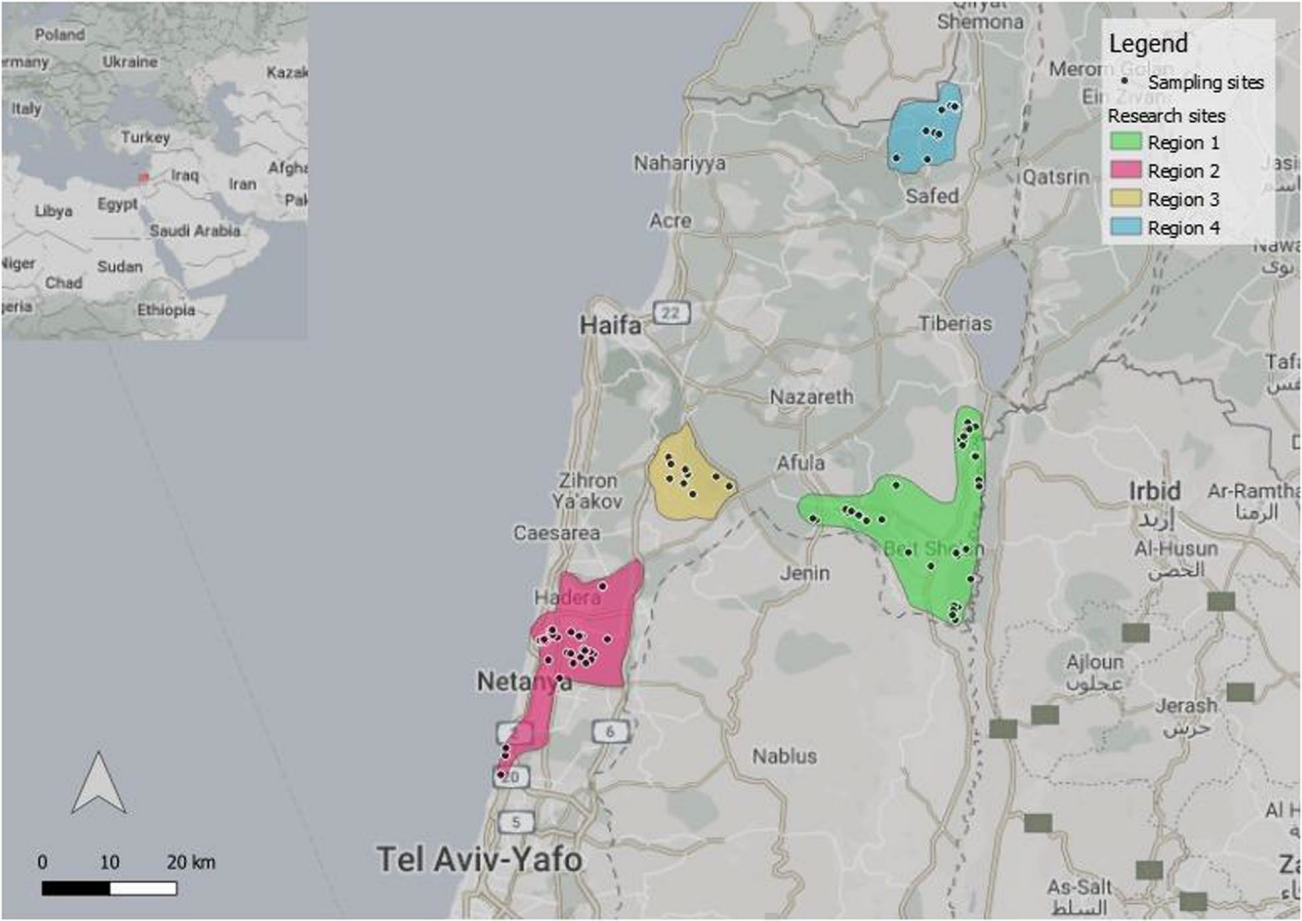
Distribution of GJ sampling regions on a map of center-north of Israel: Beit-Shean valley (Region 1; ~ 347 km^2^), Ha-Sharon (Region 2; ~ 258 km^2^), Menashe Heights (Region 3; ~ 115 km^2^) and the Upper Galilee (Region 4; ~ 100 km^2^)

In Israel, the GJ population is maintained regularly under the predator control activities of the Israel Nature and Parks Authority (INPA). Culling of the GJ is carried out mainly for rabies control, pathogen surveillance and prevention of agricultural damage. GJ sampling was performed almost exclusively, during culling activity of INPA rangers, in four different geographical regions in Israel (Fig. 1): Beit-Shean Valley (1), Ha-Sharon (2), Menashe Heights (3) and the Upper Galilee (4). Beit-Shean Valley and the Upper Galilee are considered hot spots for rabies, while the Menashe Heights is considered a relative hot spot for rabies and Ha-Sharon, is considered to be free of rabies [15]. The study cohort was planned based on sample size calculation using the Slovin's formula. The calculation took into account an estimated population size of 3000–7000 GJ (based on the mean of three to eight GJ individuals for 10 km^2^ [7]) with 95% confidence interval and 10% margin of error. The ideal sample size to achieve the goals of this study was 95 GJ samples. Therefore, we aimed for cohort size of 100 specimens from the four locations.

Sampling and data collection for each specimen was conducted shortly after culling of the jackal (usually minutes and up to one hour) and included: (1) General Information: gender (male/female), estimated age (puppy to old), body mass (kg), body score (1–5; emaciated-obese) and body length (nose to the tip of tail in cm); (2) Presence of external parasites and skin disease was also noted by inspection; (3) Biological samples: Rectal swab (ESwab™; Copan Italia S.p.A, Brescia, Italy) and blood (Serum tube- VACUETTE®, Greiner Bio-One, Kremsmünster, Austria; EDTA tube- BD Vacutainer®, BD, Plymouth, UK). All samples were stored in a cooler immediately after sampling and transferred between 30 and 360 min to a deep freezer (− 80 °C) for storage until sample processing. The carcasses of GJ were transferred to the Kimron Veterinary Institute for necropsy and further diagnostic tests.

### Burden of pathogens

The blood and tissue samples obtained from the GJ carcasses were tested at the Kimron Veterinary Institute for the following pathogens:

(1) Rabies virus by means of Immunofluorescent antibody test (FAT) on brain stem, rapid fluorescent focus inhibition test (RFFIT) for detection of rabies antibody titer from assimilation of Rabies oral vaccination [46]; (2) exposure to Rabies oral vaccination test (tetracycline test from bone) [47, 48], as fluorescence in bone tissue (usually mandible); (3) Canine Distemper Virus (CDV) using PCR method on brain tissue samples [49]; (4) *Brucella* spp. using the serological tests Rose Bengal [50], Buffered plate agglutination [51] and complement fixation [52]; (5) *Leptospira interrogans* and its serovars by microscopic agglutination test (MAT) [53]; (6) *Coxiella burnetii* (Q fever) via ELISA antibody detection [54]; (7) Internal parasite detection (intestinal and diaphragmatic) using the floatation method with sugar and salts for detection of fecal and diaphragmatic parasites [55]; (8) *N. caninum and T. gondii* parasites using a complementary immunofluorescence antibody test (IFAT) for the detection of specific antibodies [8].

### Geographical data

Each sampling site was characterized for sanitation condition and annual climate measurements. Data were exported using QGIS software (QGIS.org, Version 3.10). Sanitation conditions were defined as agricultural and/or waste potential food sources to the GJ, based on expert opinion from INPA ecologist of each sampling site. The sanitation conditions that were assigned included chicken coops (A), cattle (B), Sheep and Goats (C), Fishery (D), Dairy cattle (E), Crops (F) and landfill (G).

Climate measurements included information on Annual Precipitation (mm), Annual Mean Temperature, Maximal Temperature, and Minimal Temperature was collected for each specimen using the information at the WorldClim website (https://www.worldclim.org/).

### Fecal microbiome sequencing and analysis

#### DNA extraction and sequencing

DNA extraction from fecal swabs samples was conducted using DNeasy PowerSoil (QIAGEN®, Hilden, Germany) kit according to manufacturer’s instructions. DNA extracts were subjected to two rounds of amplification to prepare the libraries for sequencing. The first amplification of 20 cycles was performed on the V3-V4 region of 16S rDNA (amplicon size ~ 300 bp) using 16S rRNA primers from the Earth microbiome project (515F: 5′GTGCCAGCMGCCGCGGT3′, 807R: 5′GGACTACHVGGGTWTCT3’), with universal adapters CS1 and CS2. The second amplification of 10 cycles was performed using the Access Array Barcode Library for Illumina Sequencers from Fluidigm. The final library concentration for each sample was determined using the Qubit (Invitrogen) and the Denovix dsDNA High Sensitivity Kit according to the Denovix kit instructions. The size of each library was determined by TapeStation analysis using the D1000 Screentape according to the manufacturer’s instructions. Sequencing of all amplicons was carried out on an Illumina MiSeq sequencing platform using a Miseq V2-500 cycle kit to generate 2 × 250 paired-end reads) (Illumina, San Diego, CA, USA).

Illumina paired-end sequence data (as FASTQ files) were-processed using the QIIME2 software package (ver. 2021.8) and its plugins [56]. Specifically, the ‘demux’ plugin was used to import the demultiplexed paired-end sequencing reads and to create the ‘artifact’ file (i.e. QIIME2 data format required for subsequent analyses). Read merging, adapter and quality trimming, identification of chimeric sequences, and clustering of sequences with 97% similarity threshold to amplicon sequence variants (ASVs) was conducted with ‘dada2’ plugin [57]. Our quality requirement for sample inclusion was raw read count per sample > 25,000, quality scores of reads > 20, and percentage of chimera sequences < 30%. Taxonomic annotations of ASVs with 97% similarity was assigned using the SILVA reference database [58] (https://www.arb-silva.de/, version: silva-138-99-nb-classifier, date of access: 28.12.2021). The final ASV table was rarefied at 6,000 sequences per sample, followed by downstream analyses including alpha and beta diversity, and differential abundance using the appropriate QIIME2 and ‘R’ plugins.

#### Fecal microbiota correlates of different host features, pathogen burden and habitat

The association of the host features, geographic origin (site of collection) and pathogen burden was compared with the GJ fecal microbiota composition.

Alpha diversity was assessed using observed ASVs, Shannon index, evenness and Faith's PD tests. Statistical significance between tested groups was assessed using the Kruskal–Wallis test. Beta diversity was assessed using the Bray–Curtis dissimilarity index, weighted-UniFrac and unweighted-UniFrac metrices, and tested for statistical significance using the PERMANOVA test. Mantel test was used to assess beta-correlation between quantitative variables with Bray–Curtis and unweighted-UniFrac dissimilarity indices. Relative abundance of specific taxa in significant beta correlations was assessed using LEfSE (Linear discriminant analysis effect size) analysis (using the R package micreco). Significant results were considered as LDA (Linear discriminant analysis) > 3. Firmicutes/Bacteroidota ratio between region groups and age-class was assessed using the Kruskal–Wallis test, while between sex the Wilcoxon test was applied. False Discovery Rate (FDR) adjustments were applied to p-values for all statistical tests.

#### Comparison of the GJ fecal microbiota with other canids

To compare internal and external effects on the GJ fecal microbiome, we used publicly available datasets of closely related wild and domestic canids. These included black-backed jackals (*Canis mesomelas,* BBJ) studied in central Namibia [16] and domestic dogs (*Canis familiari*s, DD) from an American study of epileptic dogs [19]. We downloaded 16S rRNA amplicon sequences from SRA using the SRA-download python tool (https://github.com/zheminzhou/SRAdownload). The analysis included 50 BBJ specimens from the SRA under accession number SRP044660 and 14 healthy DD (control group) specimens under accession number PRJNA612483 together with our data on 111 specimens of GJ. The analysis of the 175 sequences, as FASTQ files, was conducted similar to GJ pipeline analysis in QIIME2, that was used on the original data (see above), with trimming adjustments of the sequence control phase (DADA2) for concluding all sequences. We then assessed taxonomic abundance, alpha and beta diversity, Firmicutes/Bacteriodata ratio and LEfSE analysis using the same methods used to analyze the study dataset of GJ (see above).

## Results

### GJ specimen sampling

The sampling effort took place from 2019 to 2020 and during this time 111 GJ specimens were collected. The Distribution of sampling region, gender and age estimation is summarized in Table 1. Additional information is summarized in Additional file 1: Table S1. Average body weight of the specimens was 9.63 ± 2.27 kg (10.05 ± 2.45 for males; 9.24 ± 2.04 for females) and body length 101.14 ± 11.36 cm (101.33 ± 11.93 for males; 99.01 ± 10.78 for females). Most of the GJ specimens (92.8%) were in normal body condition and the rest (7.2%) were thin. Twelve specimens (10.8%) were infested with mites and only five (4.5%) were infested with ticks or fleas.

**Table 1:** General information of the studied GJ cohort

	Region 1 (Beit-Shean Valley) (n = 40)	Region 2 (Ha-Sharon) (n = 39)	Region 3 (Menashe Heights) (n = 16)	Region 4 (Upper Galilee) (n = 16)	Total (n = 111)
Female/male ratio (%)	21/19 (52.5/47.5)	20/19 (51.28/48.7)	9/7 (56.25/43.75)	7/9 (43.75/56.25)	57/54 (51.35/48.65)
Adult/sub-adult/juvenile ratio (%)	21/12/7 (52.5/30/17.5)	30/7/2 (76.92/17.95/5.13)	4/11/1 (25/68.75/6.25)	8/2/6 (50/12.5/37.5)	63/32/16 (56.76/28.83/14.41)

### GJ Burden of pathogens

Diagnostic tests for pathogen detection were performed on the GJ specimens at varying rates. Results are summarized in Table 2 and additional information appears in Additional file 1: Table S2. A necropsy was conducted on all GJ specimens, and abnormal pathologies were not found, except for skin disease and heavily parasitized specimens, as mentioned above. One specimen had signs of ocular and nasal discharge and later was found positive to Distemper virus. All GJ were negative to Rabies virus. Immunity to rabies virus was detected based on antibodies in 13.5% and exposure to oral vaccine (tetracycline test) in 52.8% of the GJ. Distemper virus was found in 9.7% of the GJ. Exposure to *Brucella*, Q-fever and *Leptospira* was detected in a low percentage (less than 5% each) of the GJ specimens. *Neospora* and *Toxoplasma* antibodies were found in 16% and 29.2% of the GJ, respectively. Although tested in relatively smaller numbers, 54% were found positive to fecal parasites (mostly nematodes from strongylidae family and protozoas from *Sarcocystis* genus) and 21% to the diaphragmal parasite, *Trichinella spiralis*.

**Table 2:** Burden of pathogens among studied GJ

Test type	Number of tested GJ	Number of positive cases (%)
Internal parasite detection (fecal)	37	20 (54)
Exposure to Rabies oral vaccination (tetracycline test from bone)	106	56 (52.8)
*Toxoplasma* serological test	106	31 (29.2)
Internal parasite detection (diaphragmatic)	46	10 (21)
*Nesopora* serological test	106	17 (16)
Rabies antibodies detection	96	13 (13.5)
Distemper virus detection (brain PCR test)	92	9 (9.7)
Q-fever serological test	64	3 (4.6)
*Leptospira* serological test	65	1 (1.5)
Rabies detection (immunofluorescent test)	111	0 (0)
*Brucella* serological test	56	0 (0)

### Geographic features

Food sources and sanitation conditions around 5 square kilometers of each sampling location were characterized and mapped (Additional file 1: Figure S1) indicating differences: region 1 fisheries, sheep and goats and landfill; region 2—dairy cattle, crops and chicken coops; region 3—dairy and beef cattle; region 4—chicken coops, cattle and sheep and goats.

Climate measurements around 5 square kilometers of each sampling location were retrieved from the WorldClim website (Table 3**)**. Precipitation measurements were highest in region 4 and lowest in region 1. Mean temperature was the highest in region 1 and lowest in region 4. Coldest month temperature was measured in region 4 and warmest month temperature in region 1.

**Table 3:** Climate measurements among studied regions

	Region 1 (Beit-Shean Valley)	Region 2 (Ha-Sharon)	Region 3 (Menashe Heights)	Region 4 (Upper Galilee)
Annual precipitation (mm)	372.3 ± 46.644	568.89 ± 7.02	581.75 ± 25	625.5 ± 92.13
Mean Temperature (°C)	21.77 ± 0.58	20.17 ± 0.05	19.53 ± 0.09	18.51 ± 0.61
Warmest month Temperature (°C)	35.98 ± 1.33	31.26 ± 0.16	31.16 ± 0.24	31.66 ± 0.54
Coldest month Temperature (°C)	8.40 ± 0.23	9.12 ± 0.11	8.01 ± 0.08	6.97 ± 0.57

### GJ fecal microbiome analysis

16S rRNA amplicon sequencing of fecal samples from the 111 GJ resulted in a total of 3123331 sequences after demultiplexing. Read abundances per individual ranged from 12,322 to 61,957 with an average of 28,138.12 ± 11,320.96. After completing the DADA2 pipeline, read abundance per individual ranged from 4761 to 32,262 with an average of 13,200.65 ± 4963.64. For downstream analyses we used 110 samples containing a minimum of 6000 sequences per sample after rarefaction.

#### GJ fecal microbiome taxonomic profile analysis

**Fig. 2:**
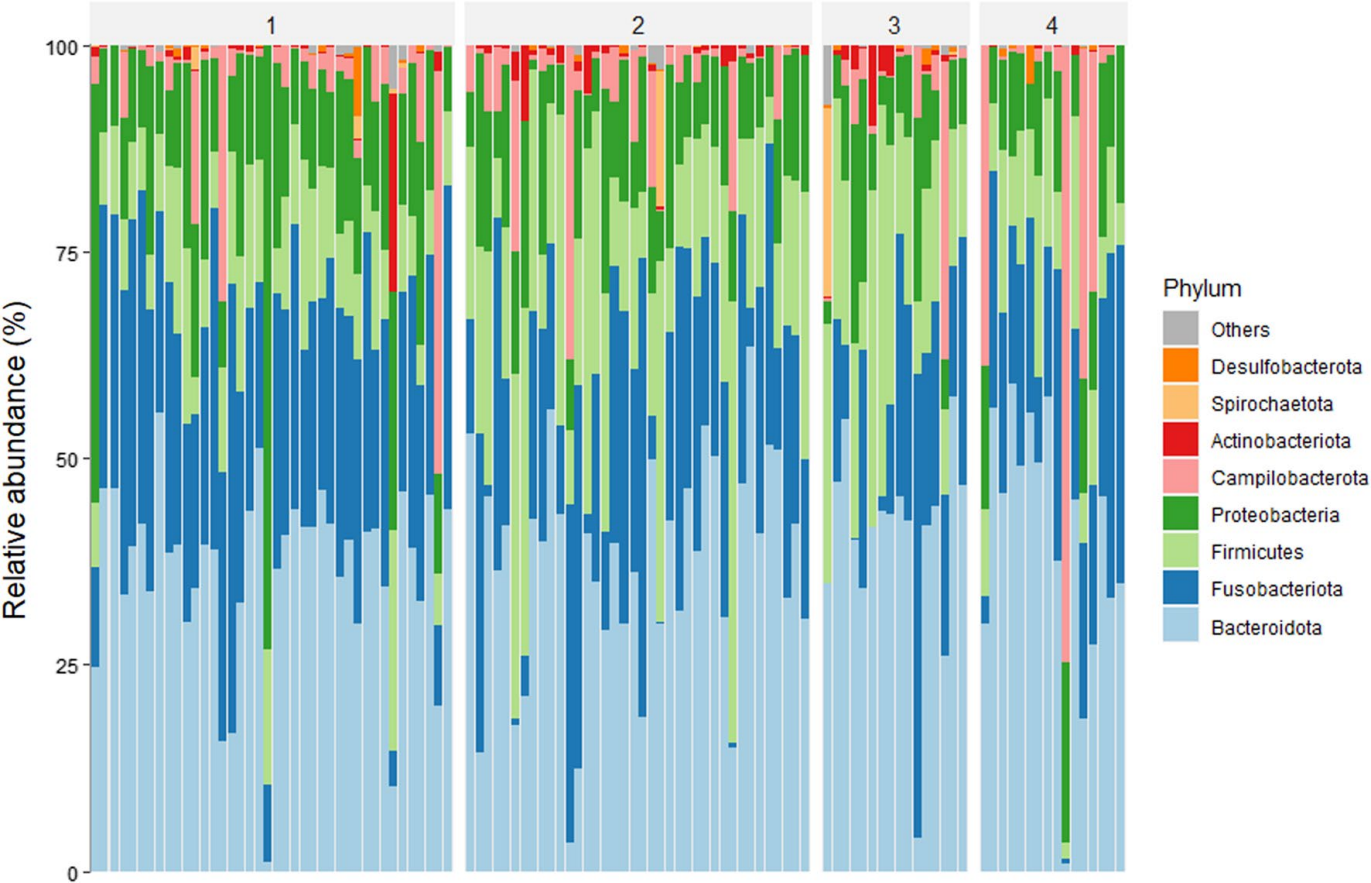
Relative abundance of top eight most abundant bacterial phyla found among studied GJ specimens, clustered by regional groups (1–4)

Bacterial taxa varied largely in their proportions between individuals; 25 phyla were found across all individuals but only 8 phyla showed abundance > 0.1% and accounted for more than 99% of relative abundance (Fig. 2 and Table 4). The most abundant bacterial phyla were the Bacteroidota (37.74%, range 1–63.57%), Fusobacteriota (24.29%, range 0–56.13%) and Firmicutes (16.53%, range 2.02–53.6%). Among them a total of 202 bacterial families were identified but only 49 were above 0.1% abundance. These families were present in almost all individuals and accounted for 97.86% of total abundance (13 families > 1% abundance; Table 5). The most abundant bacterial families were the Fusobacteriaceae (24.04%, range 0–57.07%), Bacteroidota (20.63%, range 0–44.97%), and Prevotellaceae (13.54%, range 0–41.21%).

**Table 4:** Relative abundance of leading phyla

Phylum	Mean	Min	Max
Bacteroidota	37.74	1.00	63.57
Fusobacteriota	24.29	0.00	56.13
Firmicutes	16.53	2.02	53.60
Proteobacteria	13.88	1.13	73.05
Campilobacterota	5.57	0	74.55
Actinobacteriota	0.94	0	23.90
Spirochaetota	0.43	0	22.82
Desulfobacterota	0.26	0	8.55
Unassigned	0.13	0	2.28
Other	0.24	0	13.18
Total	100.00	4.15	391.63

**Table 5:** Relative abundance of leading bacterial families

Family	Mean	Min	Max
Fusobacteriaceae	24.04	0	57.07
Bacteroidaceae	20.63	0	44.97
Prevotellaceae	13.54	0	41.21
Succinivibrionaceae	7	0	28.01
Lachnospiraceae	5.35	0	22.08
Helicobacteraceae	5.26	0	74.13
Sutterellaceae	2.59	0	9.86
Enterobacteriaceae	2.37	0	43.97
Selenomonadaceae	1.59	0	16.79
Acidaminococcaceae	1.48	0	6.1
Ruminococcaceae	1.38	0	7.97
Peptostreptococcaceae	1.25	0	7.8
Clostridiaceae	1.11	0	11.52
Other	12.46	0	473.32
Total	100.00	0	851.54

Analysis of bacterial genera revealed 437 genera, of which 59 were above 0.1% abundance and accounted for 95.36% of total abundance (Additional file 1: Table S3). The most abundant genera in the GJ were *Fusobacterium* (23.93%, range 0–55.73%), *Bacteroides* (20.82%, range 0–44.97%), *Alloprevotella* (6.98%, range 0–21.47%), *Anaerobiospirillum* (6.53%, range 0–28.17%), *Helicobacter* (5.31%, range 0–74.38%) and *Prevotella* (3.75%, range 0–40.7%).

#### Firmicutes/bacteroidota ratio analysis

The Firmicutes/Bacteroidota ratio varied greatly between individuals (Additional file 1: **Table S4**), with the mean ratio for all GJ being 0.69 ± 1.41 (ranging between 0.11–14.6). A significant difference in the Firmicutes/Bacteroidota ratio was observed between regions (*Kruskal–Wallis*; H = 17.62, df = 3, P = 0.000528), while no significant differences were observed between age-class (*Kruskal–Wallis*; H = 2.69, df = 2, P = 0.26) and sex (*Wilcoxon*; W = 1544, P = 0.844) groups.

#### Alpha diversity analysis

**Fig. 3:**
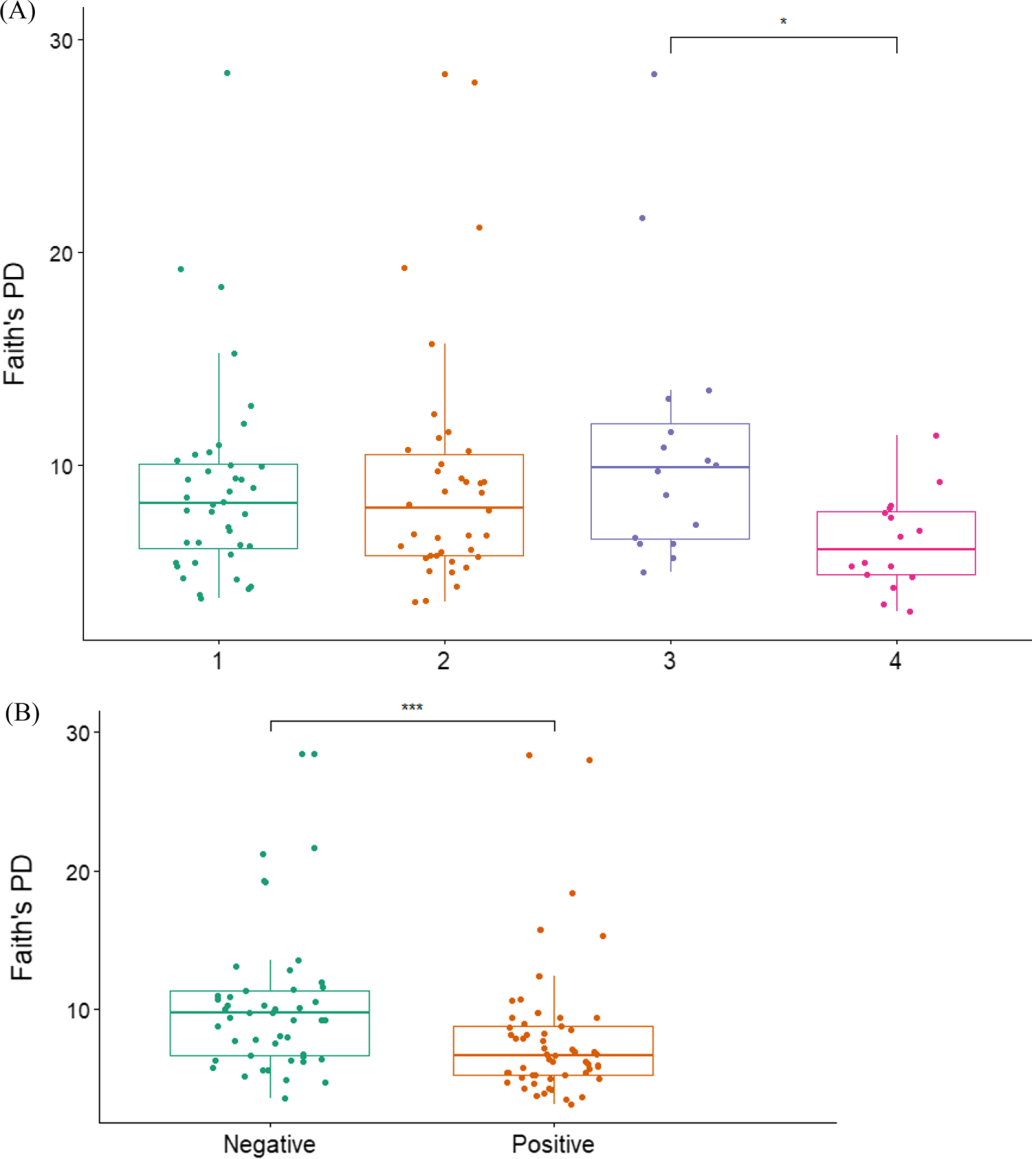
.Alpha diversity per Faith's PD **A** between regional groups (1–4); **B** between negative to positive bone tetracycline test specimens

Alpha diversity was measured using a number of metrics including Faith’s phylogenetic diversity, Shannon index and evenness. Although significant differences were not found between all regional groups in Faith's PD (*Kruskal–Wallis*; H = 9.284, P = 0.09), pairwise comparison between regions 3 to region 4 was significant (Dunn’s test, P = 0.018) (Fig. 3A). Sex, age group and body condition were not found to be significant associated with alpha-diversity. Among disease or pathogen burden, skin disease was not found to contribute to alpha diversity (Faith's PD; H = 0.221, P = 0.638). Bone tetracycline was found to be significantly associated with alpha diversity, both in Faith's PD (H = 13.217, P = 0.00028) (Fig. 3B) and evenness (H = 7.243, P = 0.007). Of studied pathogens, Canine distemper (Evenness; H = 5.53, P = 0.0186) and fecal parasites (Faith’s PD; H = 5.65, P = 0.017) (Additional file 1: Figure S2) were found as significant contributors to alpha-diversity. Sanitation conditions were not found to contribute to alpha diversity in all comparisons.

Spearman’s correlation coefficient was used to measure correlation between the quantitative measurements (GJ body and climate measurements) and alpha diversity (using the Shannon index) (Additional file 1: Table S5), but found no significant correlation (Spearman’s rho = 0.0729 and P = 0.449 for length; Spearman’s rho = − 0.0436 and P = 0.651 for weight; Spearman’s rho = 0.0287 and P = 0.7661 for annual Precipitation; Spearman’s rho = 0.0648 and P = 0.5015 for annual mean temperature; Spearman’s rho = − 0.0379 and P = 0.6941 for warmest month temperature; Spearman’s rho = 0.122 and P = 0.2043 for coldest month temperature).

#### Beta diversity analysis

**Fig. 4:**
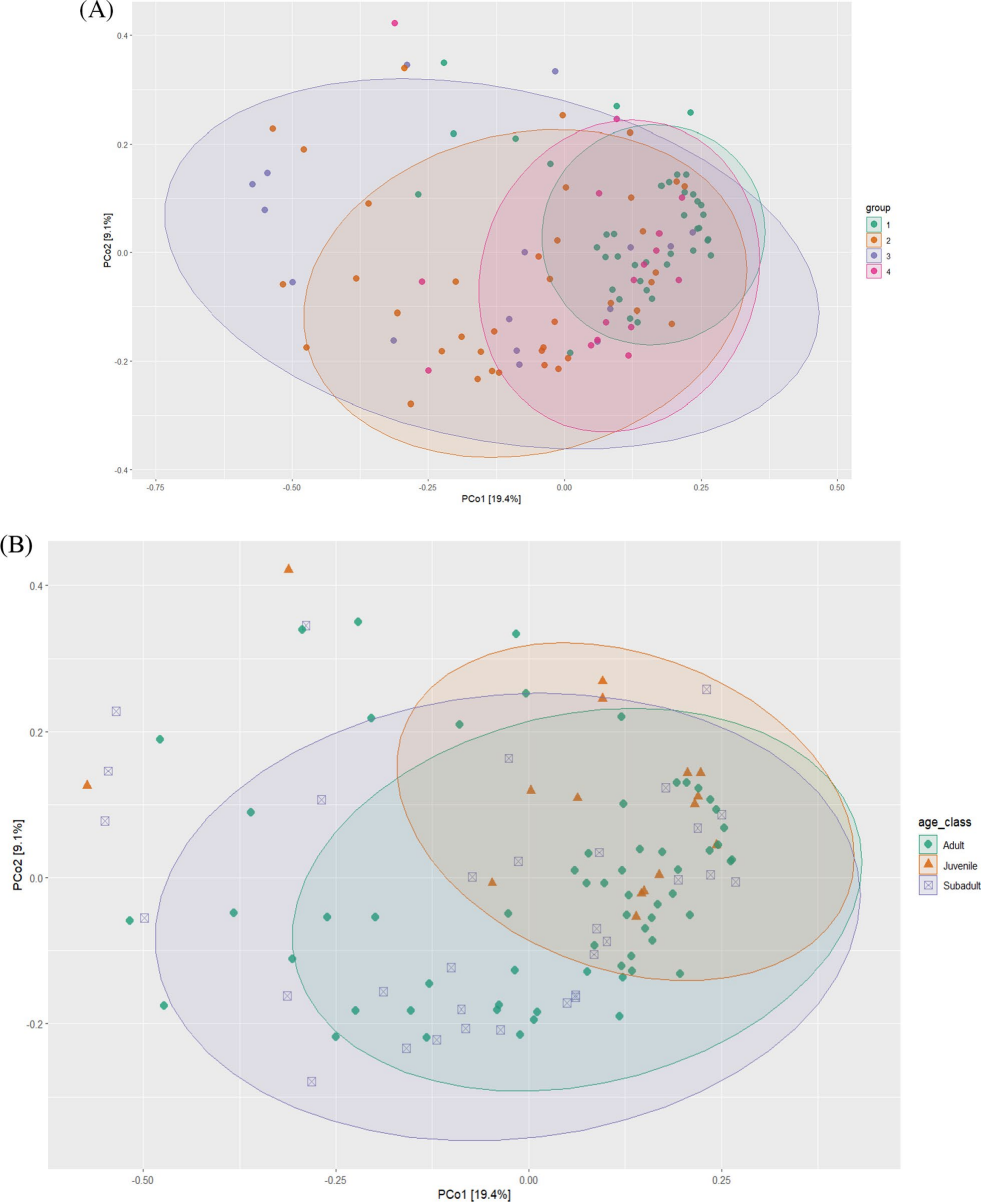
PCoA plots based on Bray–Curtis dissimilarity metric demonstrated the differences between regional groups (**A**) and age-class (**B**)

**Fig. 5:**
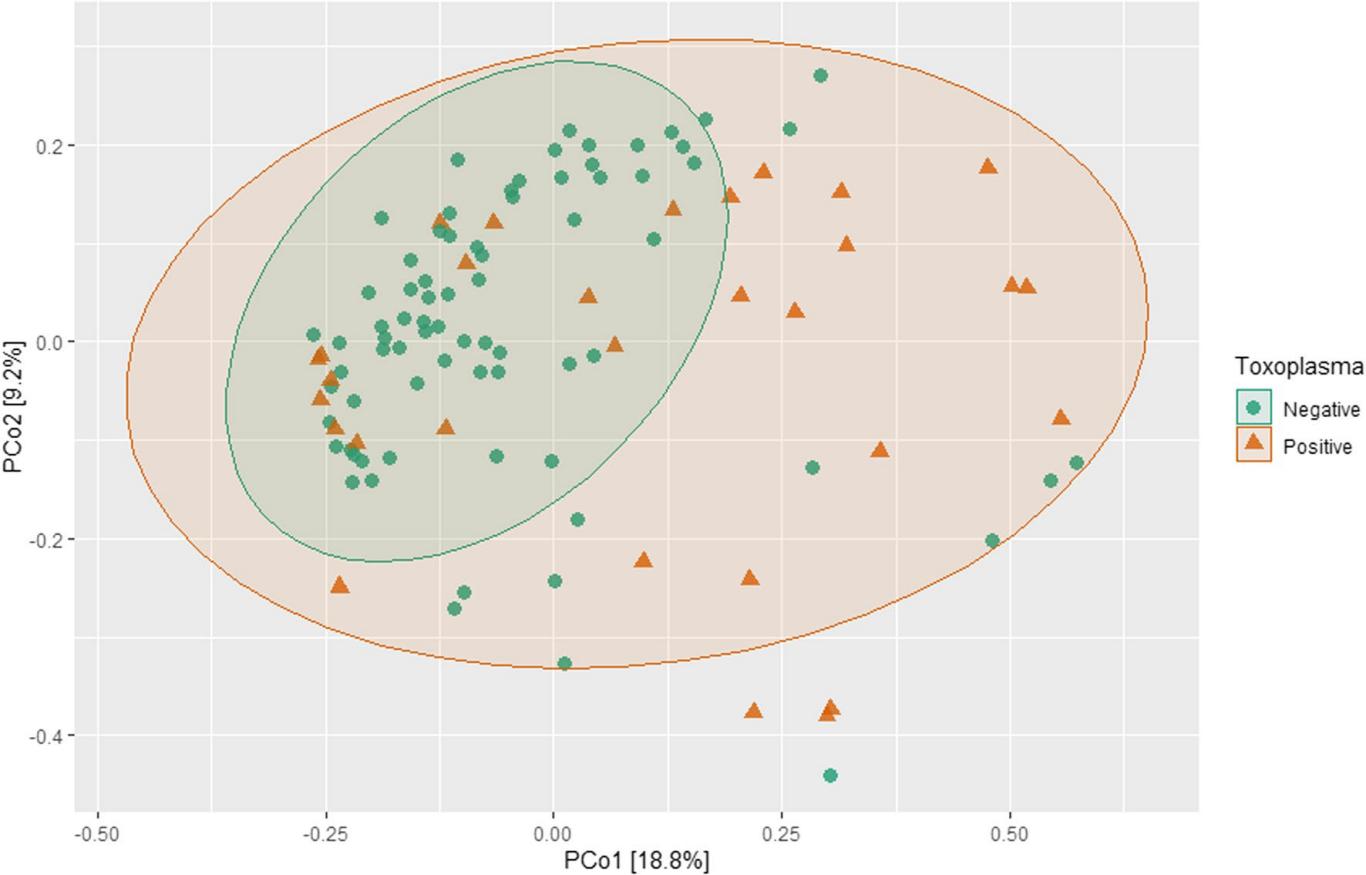
PCoA plots based on Bray–Curtis dissimilarity metric demonstrated the differences between negative and positive *Toxoplasma* specimens

Beta diversity was compared between groups using a number of metrics, including Bray–Curtis dissimilarity index, unweighted and weighted UniFrac. A significant difference was observed between region groups inBray-Curtis (F = 3.4331, P = 0.001) and unweighted UniFrac (F = 2.66, P = 0.001), and between age classes in Bray–Curtis (F = 1.6803, P = 0.014) and unweighted UniFrac (F = 2.02, P = 0.001), while no significant difference was observed for sex (Bray–Curtis; F = 0.6856, P = 0.871 and unweighted UniFrac; F = 0.71, P = 0.88)*.* Dissimilarity was demonstrated between microbial communities across regional groups (Fig. 4A) and age groups (Fig. 4B**)**. Pathogen burden was also found significant for skin disease in Bray–Curtis (F = 2.006, P = 0.014) but not in unweighted UniFrac (F = 1.45, P = 0.081), bone tetracycline in unweighted UniFrac (F = 2.072, P = 0.008) but not in Bray–Curtis (F = 1.63, P = 0.07) and *Toxoplasma* in Bray–Curtis (F = 3.025, P = 0.002) and unweighted UniFfrac (F = 2.05, P = 0.005) Dissimilarity index of positive and negative specimens to *Toxoplasma* is an example (PCoA plot Bray–Curtis; Fig. 5**)**. Plots for other significant variables (skin disease and bone-tetracycline) are shown in Additional file 1: Figure S3.

**Fig. 6:**
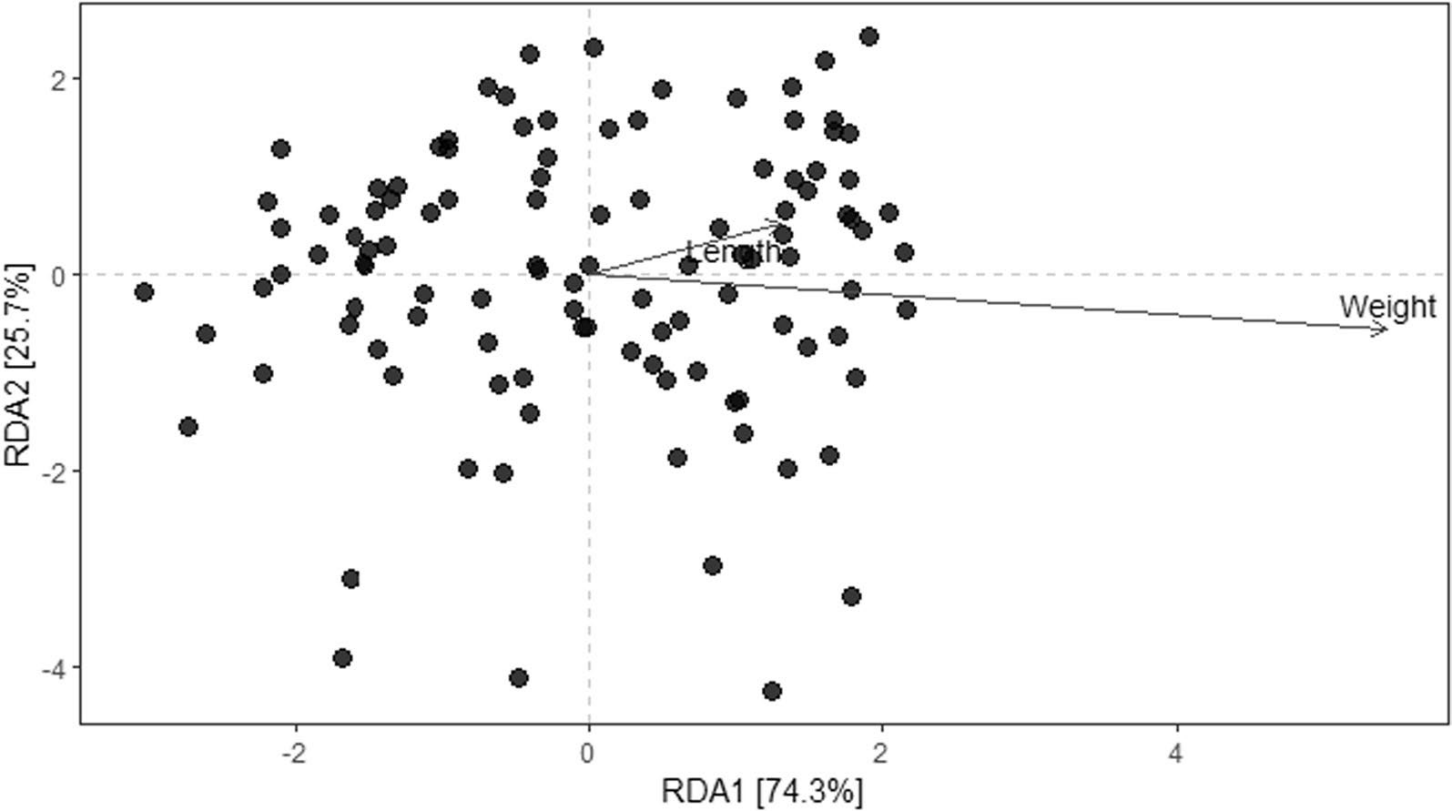
Distance-based redundancy analysis (Db-RDA) of the fecal microbiota compositions between GJ body measurements

Mantel test was used to test correlation between the quantitative measurements (GJ body and climate measurements) with beta diversity (using the Bray–Curtis and unweighted UniFrac metrics) (Fig. 6, Additional file 1: Table S6). Significant correlation was observed in the length (Bray–Curtis: Spearman’s rho = 0.146 and P = 0.018; unweighted UniFfrac: Spearman’s rho = 0.1845 and P = 0.003) and weight (Bray–Curtis: Spearman’s rho value = 0.161 and P = 0.003; unweighted UniFfrac: Spearman’s rho = 0.1811 and P = 0.003) of the specimens. Other quantitative measurements (annual precipitation, annual mean temperature, warmest month temperature; and coldest month temperature) were not found to correlate with beta-diversity.

#### Marker-gene based LEfSE analysis

LefSe analysis was performed on variables exhibiting significant findings in analysis of beta-diversity: region groups, age-class, *Toxoplasma*, skin-disease and bone tetracycline.

**Fig. 7:**
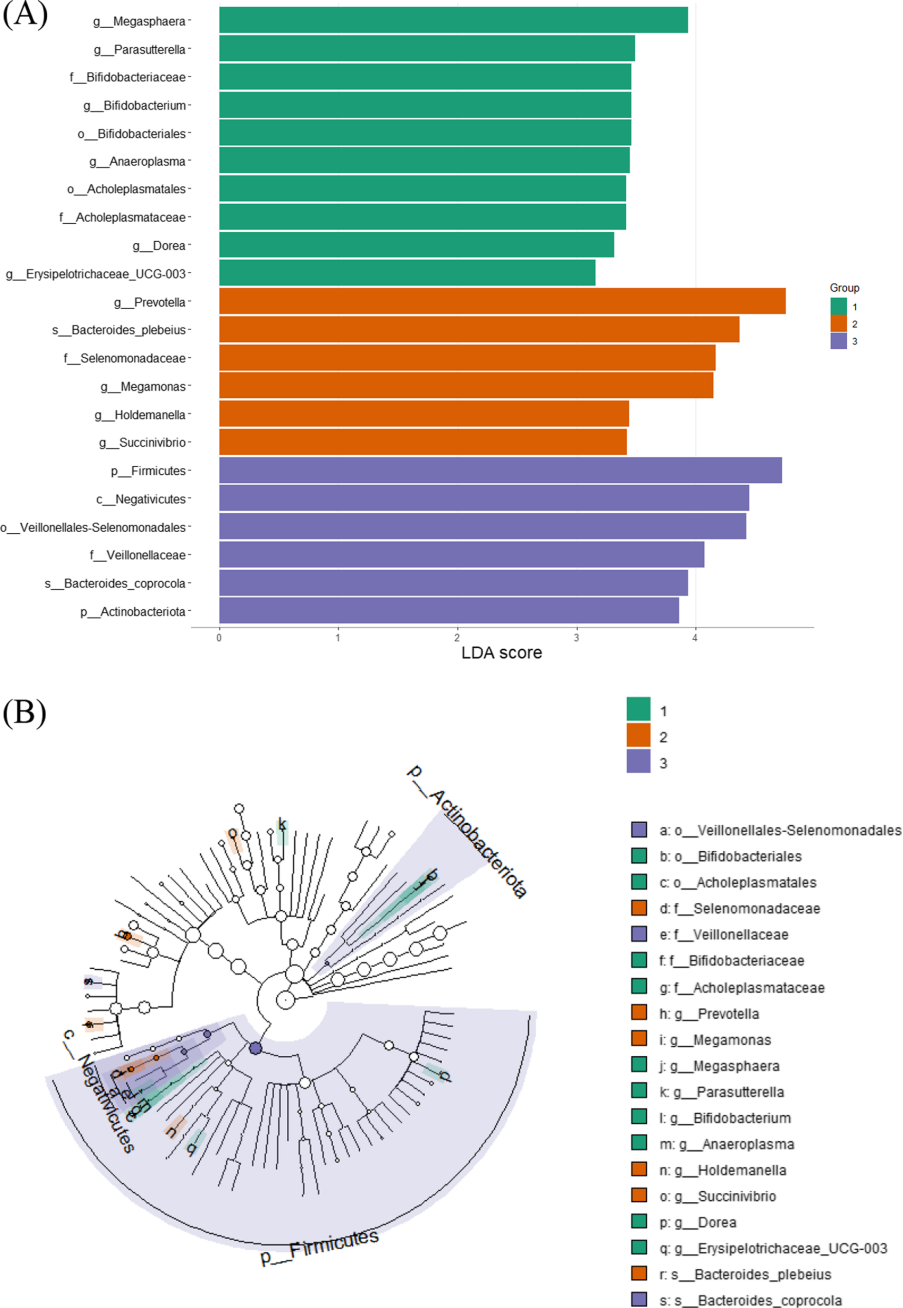
LefSe analysis regional group 1–4. **A** Score of the linear discriminant analysis (LDA, significant threshold > 3). **B** Cladogram of LEfSE results

LefSe analysis of regional groups revealed 23 significant taxa between the groups. Figure 7 demonstrates LDA scores and cladogram of LEfSe. Group 1 is enriched with *Megasphaera* genus; Group 2 with Selenomonadaceae families, *Prevotella*, *Bacteroides_plebeius* and *Megamonas* genera; Group 3 is enriched with Firmicutes phylum and Actinobacteriota, Negativicutes class, Veillonellales-Selenomonadales order, Veillonellaceae family and *Bacteroides coprocola* species. No significant taxa were found in group 4.

**Fig. 8:**
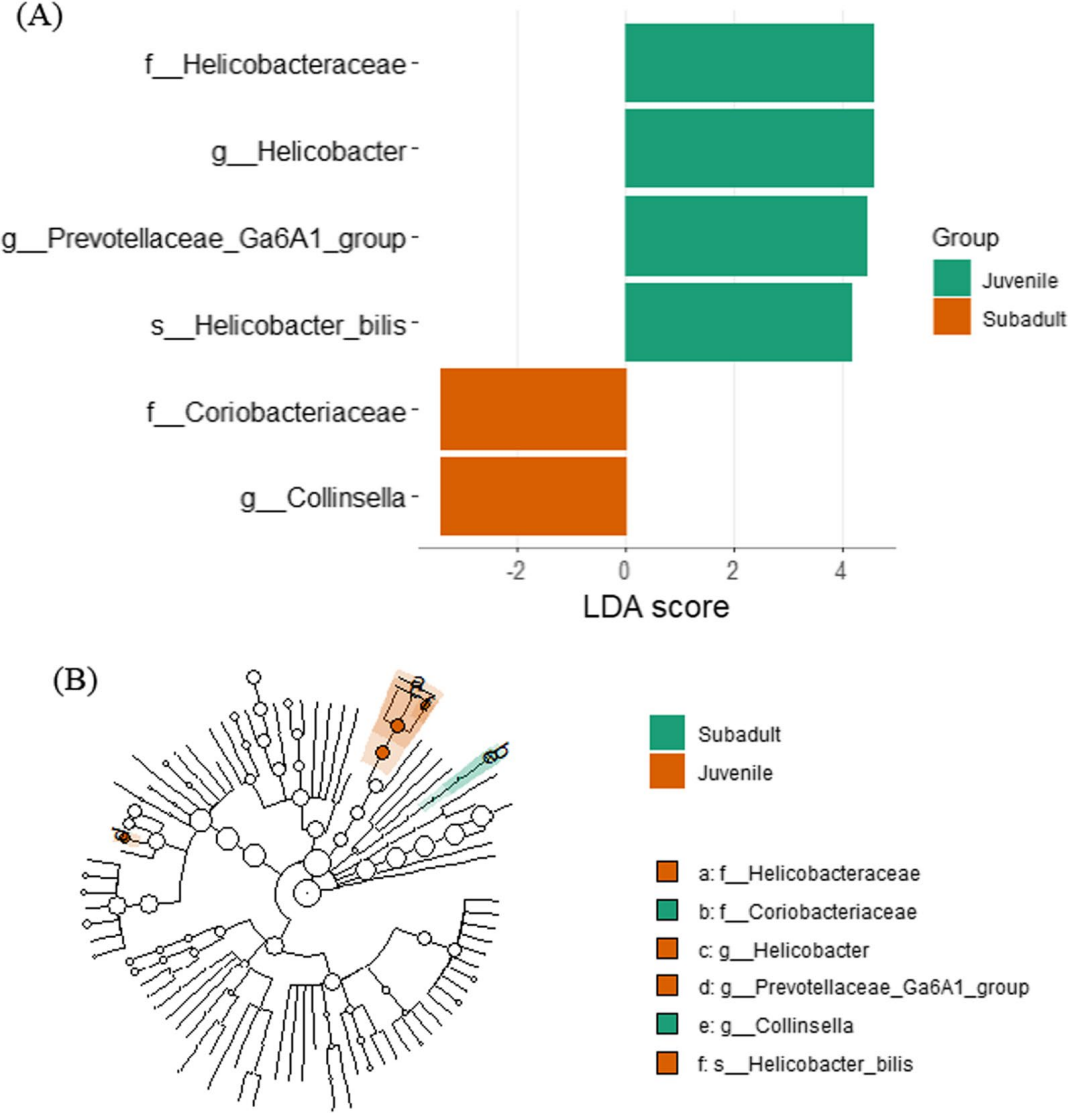
LefSe analysis for age class. **A** Score of the linear discriminant analysis (LDA, significant threshold > 3). **B** Cladogram of LEfSE results

LefSe analysis between Age-class groups specimens revealed 6 significant taxa between groups (LDA > 3). Figure 8 demonstrates LDA scores and cladogram of LEfSe. Sub-adult was enriched with Coriobacteriaceae family and Collinsella genus. Juvenile group was enriched with Helicobacteraceae family, Helicobacter and Prevotellaceae_Ga6A1_group genus and Helicobacter_bilis species.

**Fig. 9:**
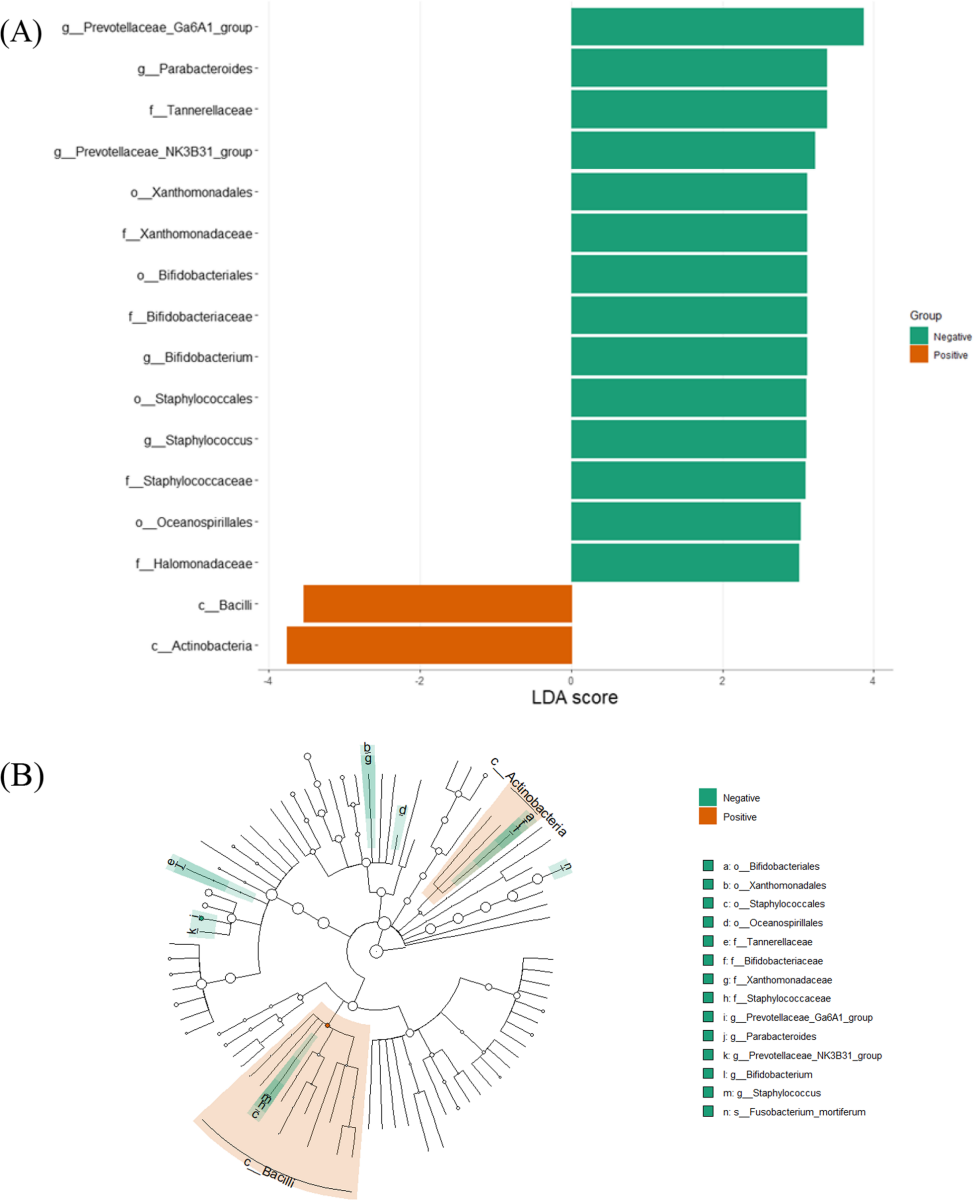
LefSe analysis for *Toxoplasma* positive and negative GJ specimens. **A** core of the linear discriminant analysis (LDA, significant threshold > 3). **B** Cladogram of LEfSE results

LefSe analysis between *Toxoplasma* positive and negative specimens revealed 37 significant taxa between groups. Figure 9 demonstrates LDA scores and cladogram of LEfSe. Positive group was enriched with the Actinobacteria and Bacilli classes. Negative group was enriched with Staphylococcales, Bifidobacteriales and Xanthomonadales classes, Tannerellaceae, Bifidobacteriaceae and Staphylococcaceae families, Prevotellaceae_Ga6A1_group, Prevotellaceae_NK3B31_group, *Parabacteroides*, *Bifidobacterium* and *Staphylococcus* genera.

**Fig. 10:**
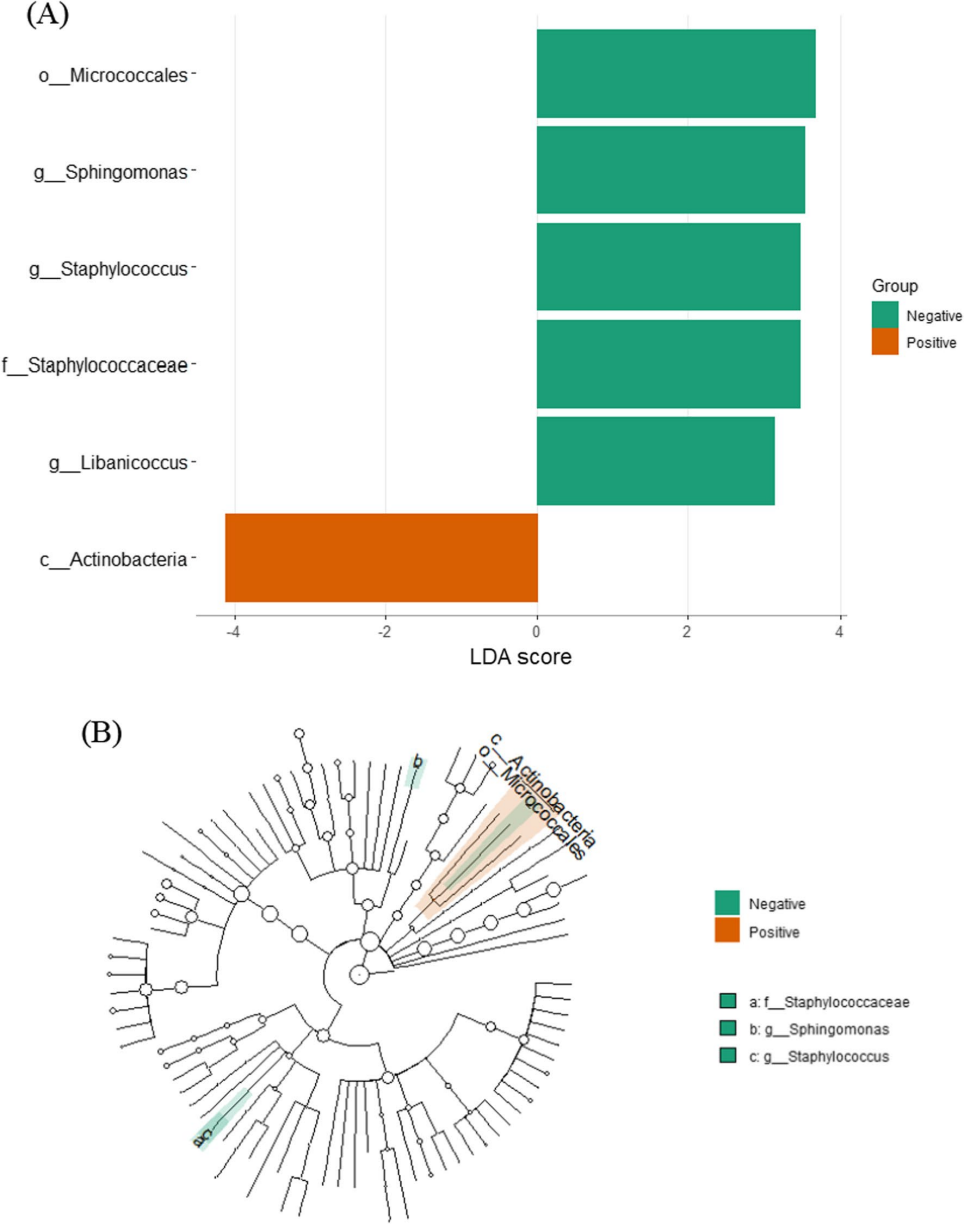
LefSe analysis for skin disease positive and negative GJ specimens. **A** Score of the linear discriminant analysis (LDA, significant threshold > 3). **B** Cladogram of LEfSe results

LefSe analysis between skin-disease positive and negative specimens revealed 36 significant taxa between groups. Figure 10 demonstrates LDA scores and cladogram of LEfSe. Positive group was enriched with Actinobacteria class. Negative group was enriched Micrococcales order, Staphylococcaceae family, *Sphingomonas*, *Staphylococcus* and *Libanicoccus* genera.

**Fig. 11:**
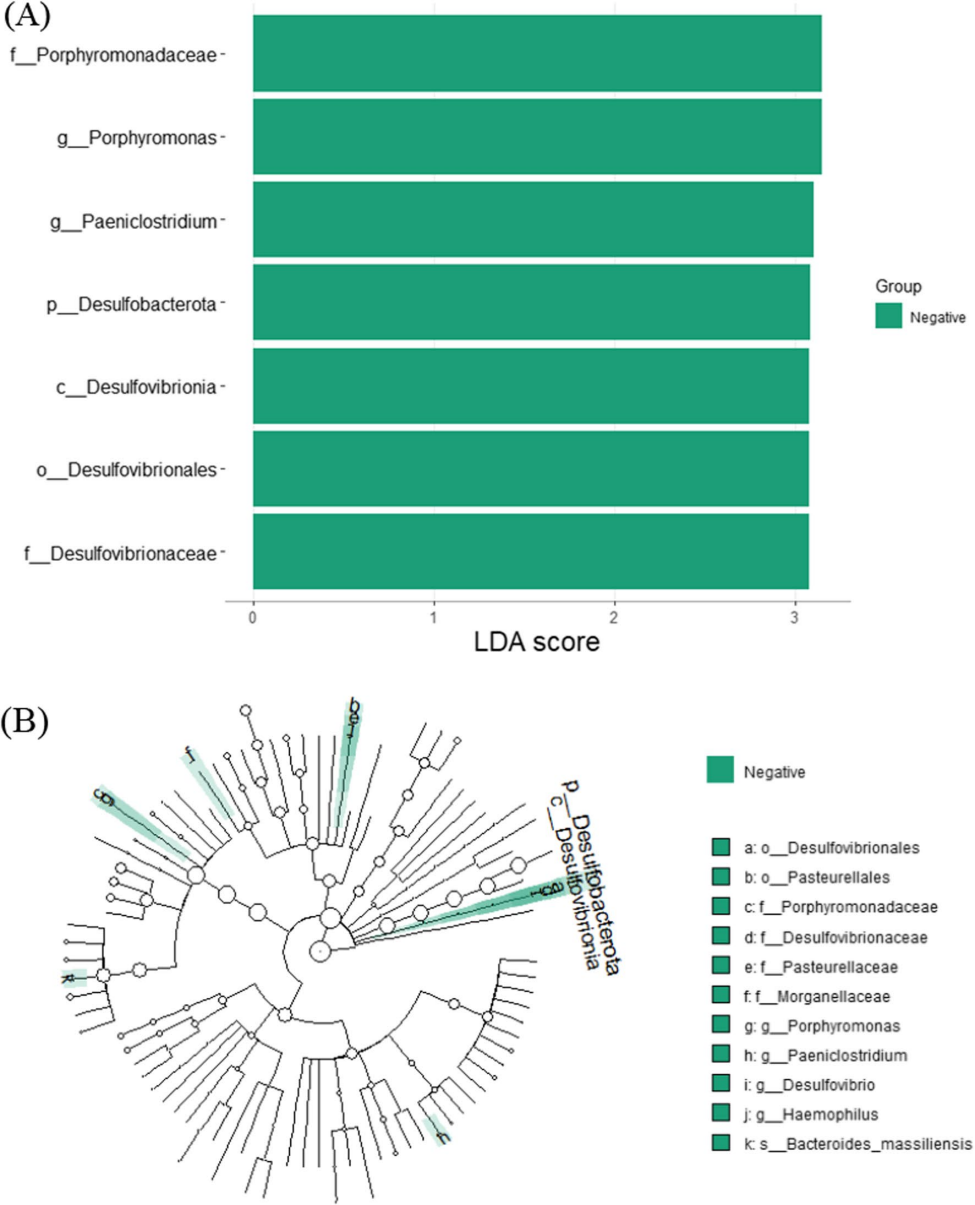
LefSe analysis for bone tetracycline positive and negative GJ specimens. **A** Score of the linear discriminant analysis (LDA, significant threshold > 3). **B** Cladogram of LEfSE results

LefSe analysis between bone-tetracycline positive and negative specimens revealed 24 significant taxa between groups. Figure 11 demonstrates LDA scores and cladogram of LEfSe. According to LDA scores, the positive group was not enriched in specific taxa. Negative group was enriched with Desulfobacterota phylum, Desulfovibrionia class, Desulfovibrionales order, Porphyromonadaceae and Desulfovibrionaceae families, *Porphyromonas*, *Paeniclostridium* and *Lachnoclostridium* genera.

#### Comparison of fecal microbiome of golden jackals, black-backed jackals and domestic dogs

**Fig. 12:**
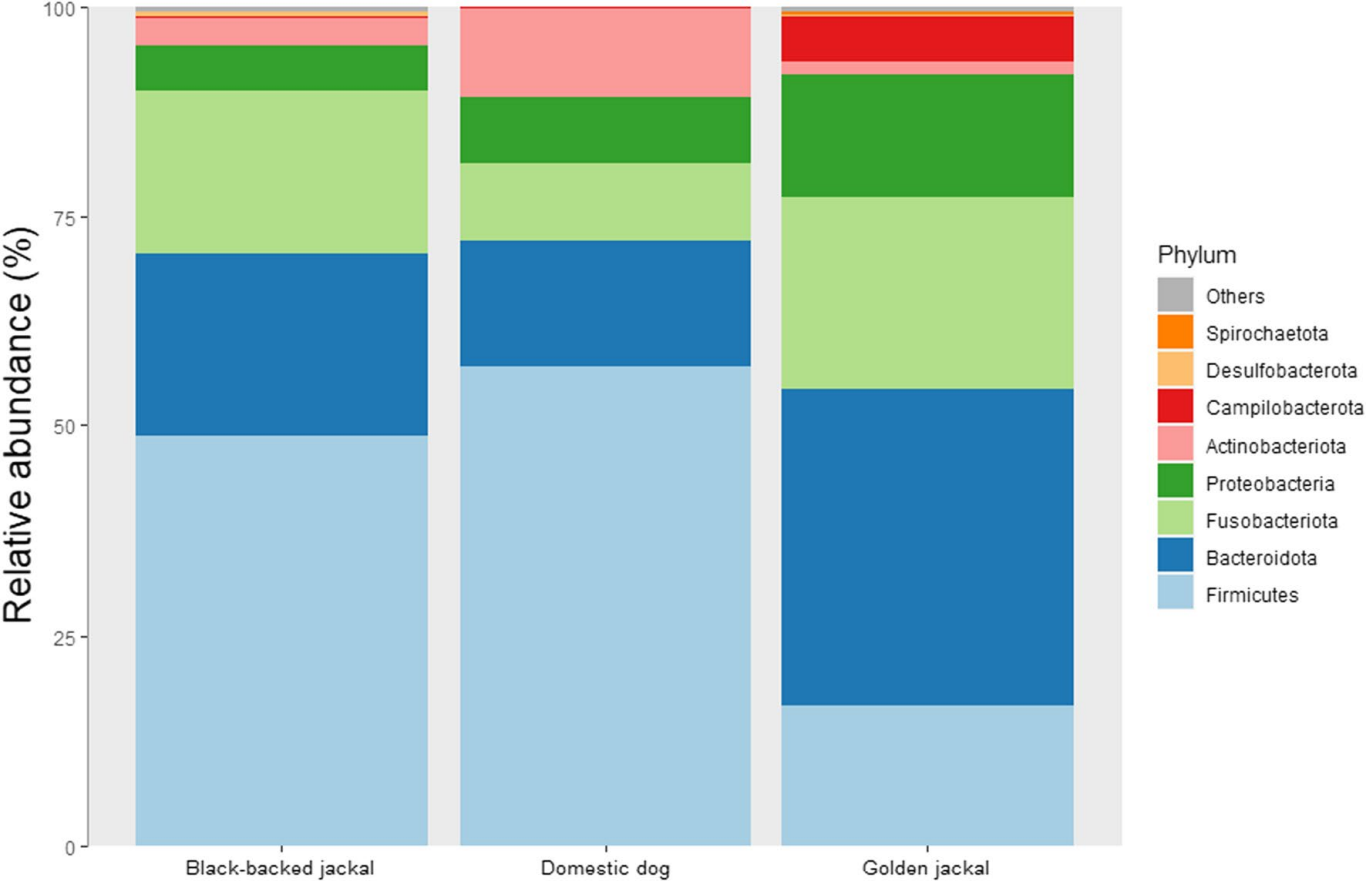
Phyla level relative abundance of core gut microbiome of the Golden jackal (GJ), Black-backed jackal (BBJ) and Domestic dog (DD)

A total of 175 specimens were used for this comparison of fecal microbiome: 111 GJ, 50 BBJ and 14 DD. Core relative abundance at phylum level (Fig. 12) demonstrates similar phyla distribution between species, with dominance of Bacteroidota (37.74%) in the GJ and Firmcutes accounts for about 50% in the BBJ and DD. Another dominant phylum in the GJ is the Campilobacterota (5.3%) and that accounts for less than 0.25% in the BBJ and DD. In the DD Actinobacteriota was found to be dominant (10.57%) compared to 3.14% in the BBJ and 1.49% in the GJ.

We compared Firmicutes/Bacteroidota ratio between the three canids species. The highest ratio was found in DD of 62.7 ± 136.68 to the BBJ with 17.96 ± 51.47 and the lowest in GJ of 0.69 ± 1.41. A significant difference was found between all the canids species (Kruskal–Wallis; H = 86.23, df = 2, P. < 0.0001) but not between BBJ to DD (Wilcoxon; H = 212; P = 0.06).

**Fig. 13:**
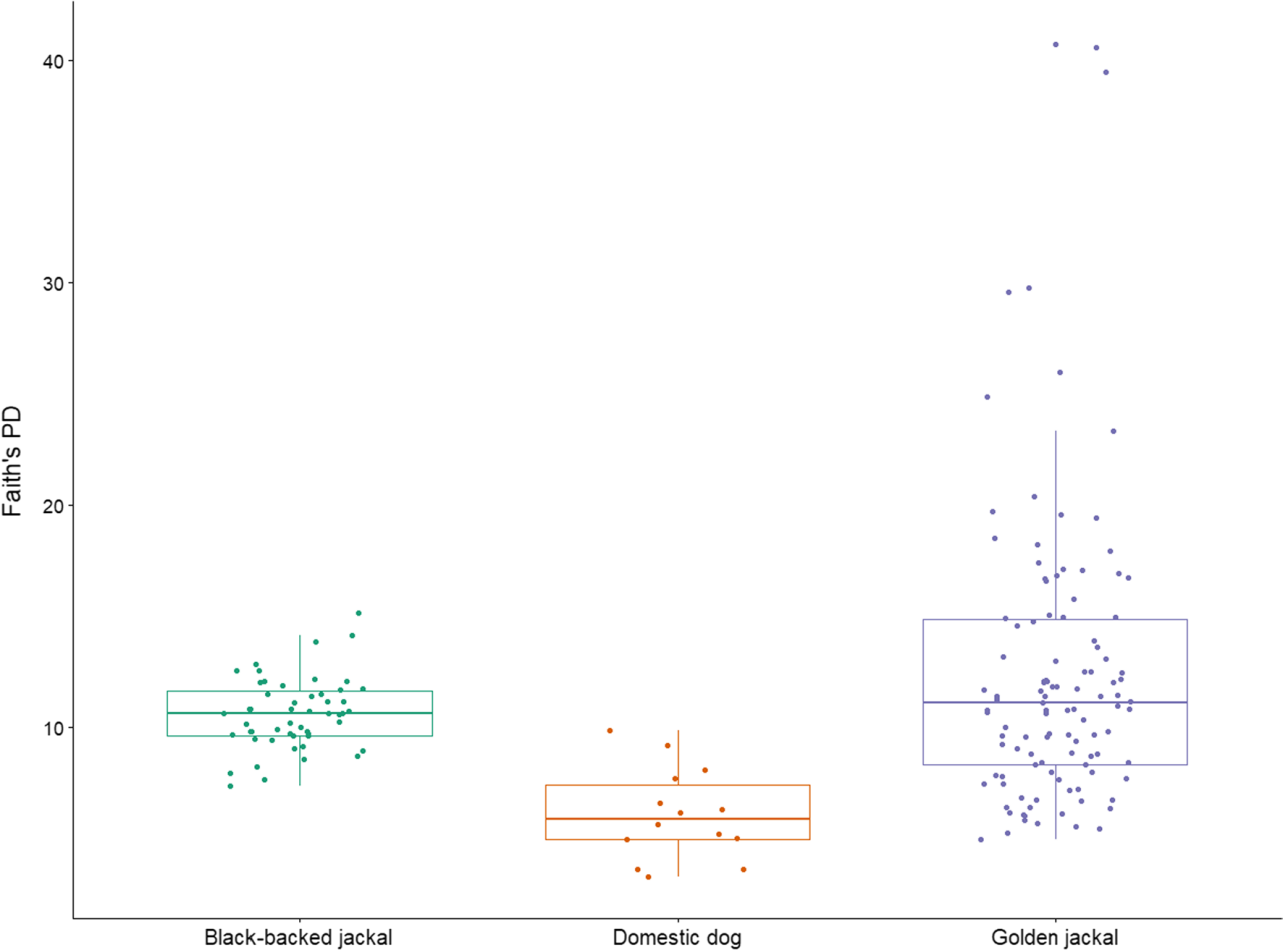
Alpha diversity in Faith's PD between Golden jackal (GJ), Black-backed jackal (BBJ) and Domestic dog (DD)

Alpha diversity analysis between the three species (Fig. 13) revealed significant differences between all groups together (Faith's PD; H = 26.27, P < 0.0001). Faith's PD pairwise analysis revealed that BBJ to GJ was not significant (P = 0.53).

**Fig. 14:**
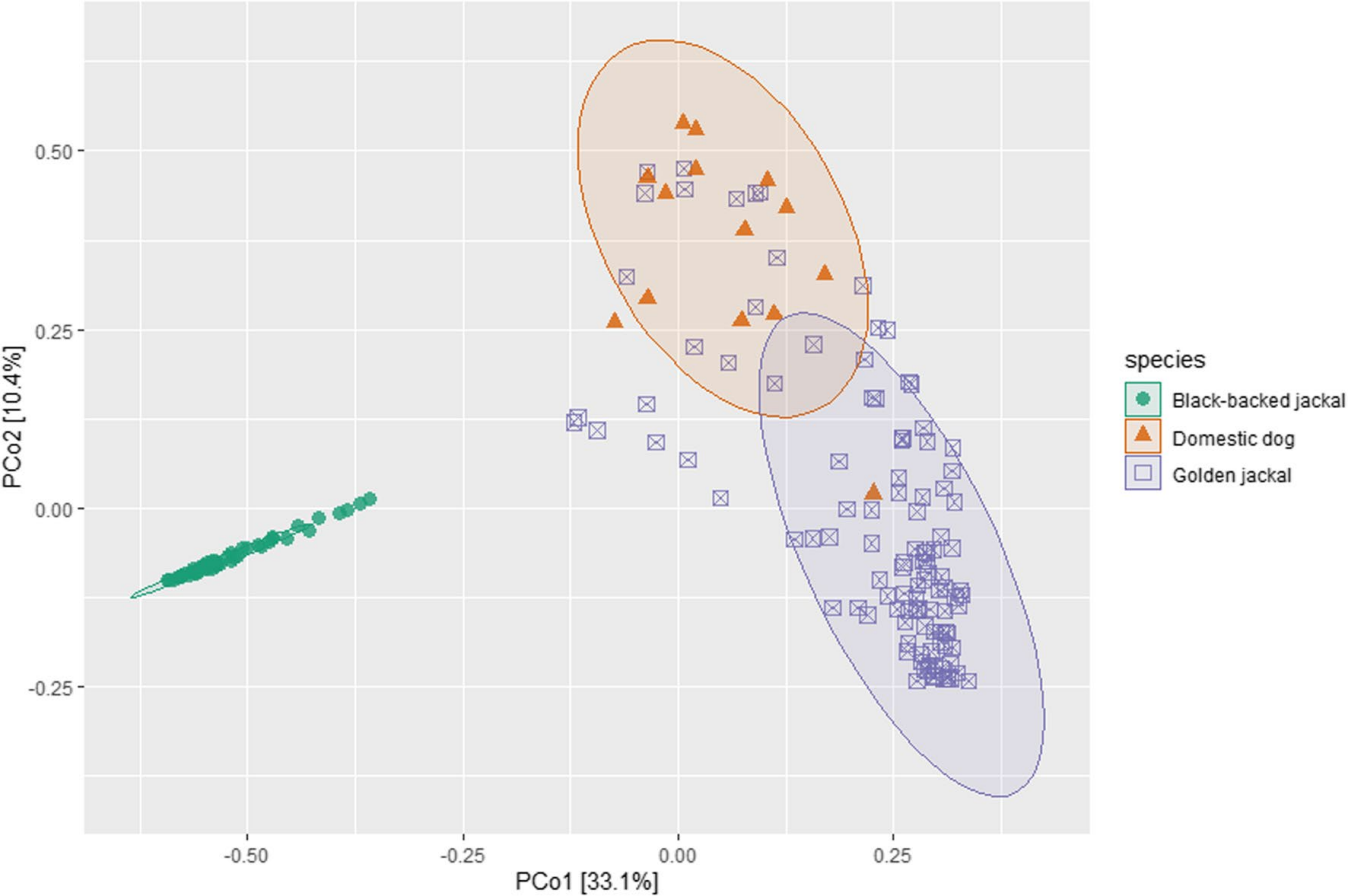
PCoA plots based on Bray–Curtis dissimilarity metric demonstrated the differences between Golden jackal (GJ), Black-backed jackal (BBJ) and Domestic dog (DD)

Beta diversity analysis revealed significant differences between all species (Bray–Curtis; F = 45.36, P = 0.001). Figure 14 PCoA plot demonstrates Bray–Curtis dissimilarity index of the three species.

**Fig. 15:**
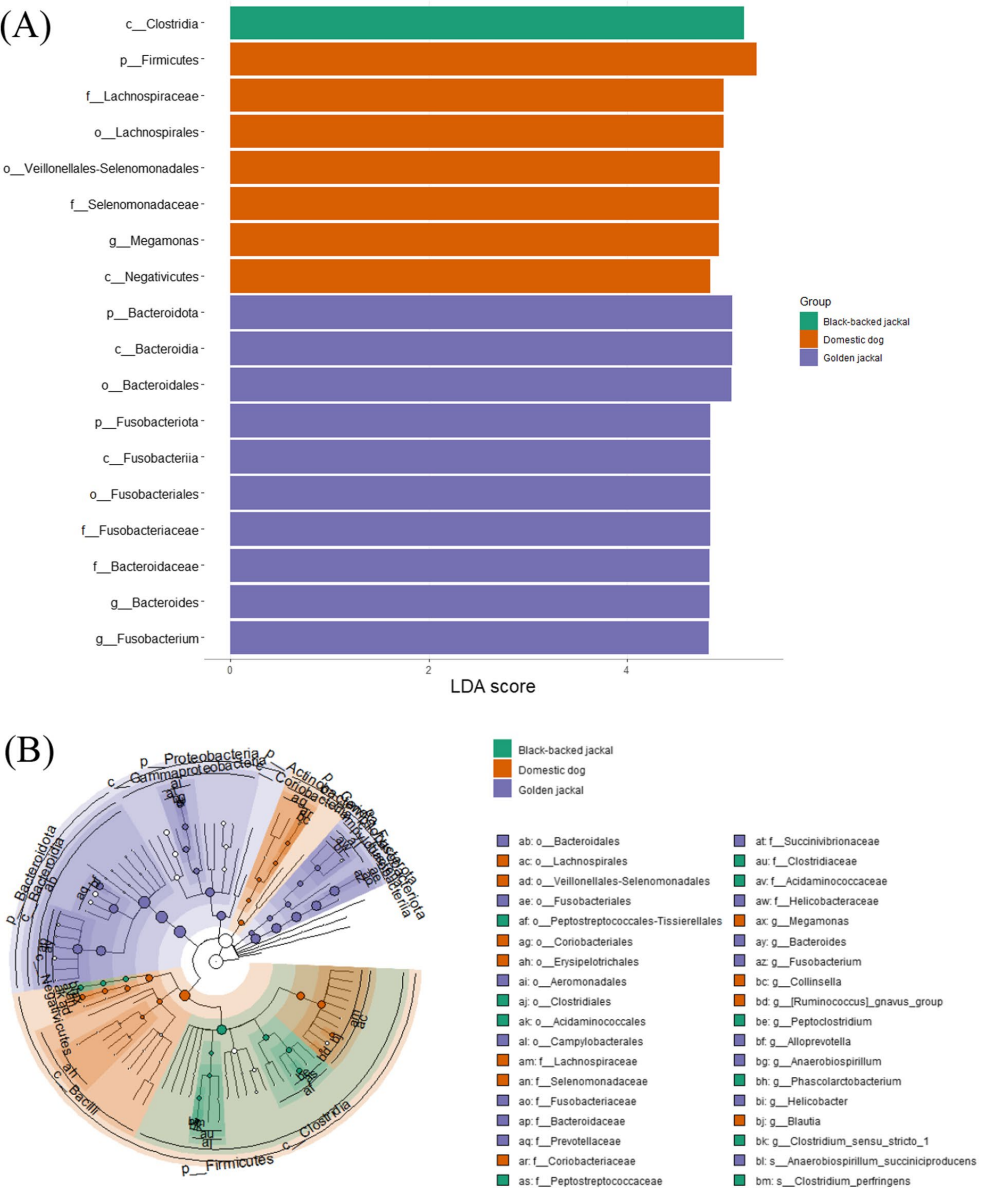
LefSe analysis between Golden jackal (GJ), Black-backed jackal (BBJ) and Domestic dog (DD). **A** Score of the linear discriminant analysis (LDA, significant threshold > 4.7). **B** Cladogram of LEfSe results

LEfSE analysis between the three species revealed 188 significant taxa (Fig. 15). In high threshold of LDA (> 4.7), The BBJ is enriched with Clostridia class. The DD was enriched with Firmicutes phylum, Negativicutes class, Lachnospirales order, Selenomonadaceae and Lachnospiraceae families and *Megamonas* genus. The GJ was enriched with Bacteroidota and Fusobacteriota phyla, Fusobacteriia and Bacteroidia classes, Fusobacteriales and Bacteroidales orders, Bacteroidaceae and Fusobacteriaceae families, *Fusobacterium* and *Bacteroides* genera.

## Discussion

In the present study, we describe novel data of fecal microbiota of free-ranging GJ. We found that Bacteriodota was the dominant phylum followed by Fusobacteriota, Firmicutes, Proteobacteria and Campilobacterota. Other representatives of the canidae family have shown versatile composition of the dominant phyla. Among the domestic dog’s fecal microbiome usually Firmicutes is considered as the main dominant phylum [59–61]. Similar proportions of fecal microbiome were found in the comparison between Black-backed jackal data [16] and domestic dogs [19]. In both Coyote (*Canis latrans*) and Red foxes (*Vulpes vulpes*) Proteobacteria were found to be dominant on Firmicutes and Bacteriodota [34] while in captive Red Wolf (*Canis rufus****)*** the dominant phyla were Firmicutes, Bacteriodota and Fusobacteriota [35]. Unfortunately, most of the canids species are prone to anthropogenic effects. Furtheremore, the GJ is known as generalist and synanthropic species that accompanies human settlements and its diet composition is dominated by human products [1]. Hence, we believe the high abundance of Bacteriodata in the GJ fecal microbiome composition is related to carbohydrate and fiber rich diets, similar to the findings in dogs [62, 63]. This suggests that our findings may reflect an adaptation of the GJ fecal microbiome to the human settlements.

The Firmicutes to Bacteriodata ratio (F/B ratio) was mainly investigated in humans [64–66] but also in animals. Increased F/B ratio has shown to correlate to obese humans and animals [39, 67]. F/B was also associated with energy harvest measurement, as a higher ratio was found in females of howler monkeys [40] and BBJ [16]. Our analysis of the GJ did not found any correlation between sex and age-class and F/B, but such correlation was found across regional groups. This may be due to different food sources or due to the relatively higher abundance of exposure to Bacteriodata compared to Firmicutes in the GJ. When compared to DD and the BBJ, the GJ was found to be with the lowest ratio. Furthermore, Bacteriodata phylum members found related to a positive interaction with intestinal immune system and intestinal barrier [68], suggesting this finding may reflect an adaptation of the GJ fecal microbiome to the human settlements. Future studies should assess the effect of different food sources on the GJ F/B ratio.

Microbiome variation can be influenced by the environment and host variability. In this study, we analyzed a variety of environmental and host traits with the GJ fecal microbiome. Several wild animal studies have found microbiome variation between different geographic populations [41, 69, 70] but not in the BBJ [16]. In our study we found an impact of research sites on GJ fecal microbiome. The effect was demonstrated mainly by beta diversity and less by alpha diversity. This effect can be due to geographic distance, different diet in each geographical area or due to host genetic segregation.

In this study we tested other environmental variables against the GJ fecal microbiome. First, we tested annual temperature and precipitation, but no correlation to the fecal microbiome was found. In the big-horn sheep (*Ovis canadensis nelsoni*) no correlation was found between multi-parameter variables including precipitation [69]. In red squirrels (*Sciurus vulgaris*) gut microbiota composition was strongly affected by seasonal dietary changes [70]. Since our cohort was sampled independently of seasons and not repetitively, we assume the effect of environmental variables could be underestimated. The effect of different diet on gut microbiome is well studied in human [71, 72] but less in wildlife. In Hooded cranes (*Grus monacha*) diet changes with seasons and correlates with gut microbiome [73]. Another environmental variable that was tested is sanitation conditions based on possible diet source to GJ that can be attributed to the fecal microbiome. We did not find any such correlation in this analysis and we hypothesize that the multiple food sources together with the GJ being a generalist species cause this effect to diminish.

Host traits of the GJ were recorded for each specimen and analyzed against the fecal microbiome. Most of the traits tested were not found to be contributing to alpha and beta diversity. Sex, for example was found to impact the gut microbiome in the BBJ [16] and elephant-seals (*Mirounga angustirostris*) [32]. In the GJ no such effect was found in alpha and beta diversities, assumingly because food foraging is carried out by both male and females [1]. On the other hand, age-class was found as a factor in shaping alpha and beta diversities in the GJ and this effect is mainly between adult to juvenile and subadult specimens. An age effect on gut microbiome was described in humans [65, 74, 75] and in lowland gorillas (*Gorilla g. gorilla*) [76] and Brandt’s voles (*Lasiopodomys brandtii*) [77]. Other host traits which reflect body measurements and general health status of the GJ were analyzed with the fecal microbiome. Length and weight measurement affected the beta diversity of GJ fecal microbiome. This effect is probably connected to age-class effect since both are usually correlated. Body condition was not found to have an effect on the microbiome. An altered gut microbiome was found in coyote with poor body condition [42]. Most of the GJ specimens were in normal body condition, suggesting that this altered state was not present. From observed pathologies as external parasitism and skin disease (as mange), skin disease was found to affect beta diversity of the fecal microbiome. Notably, in humans, gut microbiome alterations were found in atopic dermatitis, psoriasis, and rosacea [78] and dogs with atopic dermatitis have shown reduced alpha diversity compared to healthy dogs [79]. These findings suggest that alterations in the fecal microbiome of the GJ can increase the probability for being infected in skin disease such as mange.

Pathogen burden in the GJ was tested in a variety of methods. Our goal was to investigate correlation of different pathogens with the GJ fecal microbiome. All of the GJ specimens were found negative to Rabies virus and *Brucella* bacteria and therefore these were not included in the microbiome analysis. Response to ORV was measured using antibodies detection and the biomarker tetracycline. Specimens positive to tetracycline were found to have a significantly lower alpha diversity and with clear dissimilarity in beta diversity as compared to negative specimens. Mice with altered microbiome, due to antibiotics treatment, had lower humoral response to rabies vaccine, suggesting the gut microbiome impacts humoral immunity [80]. Our result for the Rabies vaccine biomarker suggests different profiles of the fecal microbiome of GJ assimilate the vaccine better than others. Further investigation is needed to clarify this effect. Canine distemper was found to affect alpha diversity of the GJ fecal microbiome with higher diversity in the infected specimens. In the giant panda (*Ailuropoda melanoleuca*), infected animals have shown increased microbial diversity and decreased relative abundance of dominant taxa compared to uninfected animals [81]. Canine distemper is a lethal disease that can infect many species of carnivores and affects mainly respiratory, nervous and gastrointestinal systems [82]. The GJ specimens were sampled during predator control and no signs of illness were noted during sampling except one specimen. It is possible that changes in the fecal microbiome diversity in infected GJ is due to progressive state of canine distemper disease, similar to the giant pandas [81].

Internal parasites were examined in feces and diaphragm. We observed that fecal parasites affected alpha diversity of GJ fecal microbiome as positive specimens had greater diversity. In the European shag (*Gulosus aristotelis*), a microbiome dysbiosis was found in heavy burden of helminth compared to a low burden [44]. Interactions between gut microbiome, helminth and the host occur constantly in the gastrointestinal tract, for example parasite mediated suppression of inflammation [83, 84]. We found that *Toxoplasma* had a notable impact on the beta diversity of the fecal microbiome. Infected mice with *Toxoplasma* have shown alterations in cecal microbiome compared to healthy mice and higher abundance of harmful bacteria, suggesting that gut microbiota has an important role in infection with *Toxoplasma* [43]. While this association did not implicate harmful bacteria in our study, it may be possible that different profiles of fecal microbiomes in the GJ can protect against *Toxoplasma* infection or, on the other hand, confer susceptibility to this parasite.

We used LEfSe algorithm method to identify taxonomic groups differences between groups of microbiome analysis [85]. Regional group analysis revealed different relative abundance of bacterial taxons between regions. *Megasphaera* was found in relatively high abundance in region 1. Megasphaera was negatively associated with diarrhea from Cryptosporidiosis [86]. We did not assess diarrhea in the GJ specimens, but cryptosporidiosis was not found in fecal examination for internal parasites. In Group 2 we observed abundance of Selenomonadaceae family that is part of Firmicutes phylum. In this family also the *Megamonas* genus was found abundant. *Megomonas* was previously found abundant in arthritic dogs compared to healthy dogs [87] and this was associated with anti-inflammatory properties and metabolic rate influence by producing acetic and propionic acids [88]. This genus abundance may also relate to the F/B ratio changes between group locations, as discussed above. *Prevotella*, commonly abundant in human microbiome, was also found abundant in group 2. *Prevotella* is related to western diet, but some representatives consider potential pathogens [89]. This abundance may be related to GJ proximity to human, or incidental discovery. Group 3 was also enriched with Firmicutes representatives similar to group 2. With the addition of Actinobacteriota abundant in the group. This phylum is accounted as beneficial to gut homeostasis in humans [90]. *Bacteroides coporcola* was also abundant, and species belonging to *Bacteroides* genus produces extracellular enzymes that assist in the breakdown of complex plant polysaccharides as cellulose and hemicellulose, as well polysaccharides like mucopolysaccharides and by that produce valuable nutrients and energy for their host [91]. Such abundance can aid the generalist characteristics of the GJ, consuming a variety of human products and waste. LEfSe analysis for *Toxoplsama* seropositivity, revealed abundance of Bacteroidota, Firmicutes, Proteobacteria and Actinobacteriota representatives in the seronegative group. *Bifidobacterium* from *Actinobacteriota* found in higher abundance in the negative group, and that associated with dysbiosis when in lower number and higher disease activity in human autoimmune disease [92]. Therefore, relative abundance of *Bifidobacterium* may have protective effect on immune mechanisms. In *Toxoplasma* seropositive group, the Bacilli class (Firmicutes phylum) was found abundant. In dogs, abundance of several genera of Firmicutes, suggested to indirect effect the infection of parasites like Giardia [93]. LEfSe analysis for presence of skin disease revealed presence of Actinobacteriota in the positive for skin disease. Other representatives of Actinobacreiota were found also in the negative for skin disease, especially the gut protective taxon, *Bifidobacterium* [94]. We can hypothesize that other Actinobacteriota genera, which were not assigned using LEfSe analysis, could affect the gut microbiome to be prone to skin disease infections. Another class that was found in skin disease cases were the Bacilli from Firmicutes phylum, that may found in higher proportion in psoriatic disease in human [94]. LEfSe analysis for biomarker tetracycline revealed only taxons in the negative for tetracycline group. In those taxons is the Porphyromonadaceae, Desulfovibrionaceae and Peptostreptococcaceae families were with higher proportions. In rhesus macaques [95] and a marked alterations was found after vaccination of HIV-1. Our profile of bacterial taxons in the negative for rabies oral vaccine biomarker can affect our understanding of immune response to the rabies vaccine.

Our comparison of GJ, BBJ and DD revealed significant differences in almost all comparisons. Alpha diversity comparison shows similar diversities between GJ to BBJ and both are different than DD. Beta diversity found significant differences between all canids, and BBJ even more distinct. This differences likely reflect the biology, genetics and geography of those canids. Although GJ and BBJ have similar omnivorous feeding behavior, fecal microbiome differ markedly, this may be due to GJ relying on human waste and agricultural products more than the BBJ [1, 14]. In LEfSe analysis between the three canids, we saw again the relatively low proportion of Firmicutes and high Bacteriodata in the GJ and the opposite in the DD and BBJ. From Firmicutes, Clostridial representatives are frequent both in DD and the BBJ, which are associated with a high protein diet [96]. In the GJ Fusobacteriota was also abundant, similar to dogs eating raw food [97] and wolves (*Canis lupus*) [98, 99]. However, as these samples were derived from different projects, we cannot rule out technical differences and findings should be corroborated by future studies.

There are several possible limitations to this study. The GJ is a versatilespecies which is nocturnal predator and fairly mobile and thus sample collection is difficult to perform, thus limiting cohort size. Nevertheless, our cohort did achieve the required sample size per power calculation. Our study was a point prevalence study in culled animals and not a longitudinal study so microbiota dynamics over time could not be tested. The lack of multiple sampling time points could have resulted in bias owing to changed diet, human food sources and seasonal effects. We did not track the animals thus could ascertain there was no movement between regions. However. We find this unlikely since past large scale studies of radio collared GJ in Israel never recorded such a movement and established the home range of GJs about 10 km^2^ and few as 1 km^2^ when roaming near human settlements [7]. Another possible bias is that our sampling was not systematic and thus resulted in different age distributions. We tried to control these limitations by a relatively high number of specimens and sampling throughout all seasons. Additionally, we compared our data to published datasets of microbiota of closely related wild and domestic canids. However, such comparison, should be interpreted with cautious due to differences in time, geographic location and sequencing methods. Another limitation is the use of 16S rRNA amplicon sequencing compared to whole genome metagenomics that could broaden our spectrum of knowledge and understanding of the GJ fecal microbiome by performing strain-level metagenomics and reconstructing metabolic pathways. Furthermore, we tested for a variety of pathogens using different methods which are well accepted but may be limited in their sensitivity or specificity as compared to advanced molecular and genomic methods.

## Conclusion

Our knowledge of wild species microbiota is still very limited despite a growing number of studies being published. Here we report novel data of the GJ fecal microbiota and its relation to host traits, pathogen burden and epidemiological characteristics. We found associations between fecal microbiota to many of the variables tested. The proximity of the GJ to human settlements together with our findings, can help to improve the understanding of human to wildlife interface. This can be underpin improved rabies surveillance through better targeting of the oral vaccine and better understanding how human waste and products can influence the pathogen carrier capabilities of the GJ. Further studies should focus on the role of host genetics of the GJ, and its effect on shaping the fecal microbiome. Moreover, employing whole genome metaganomic sequencing could further explore the interaction between the GJ, other animals and humans.

### Supplementary Information

**Additional file 1**. **Figure S1.** Sanitation conditions across sampling regions (1-4). **Table S1.** General information of GJ specimens. **Table S2.** Pathogen burden of GJ specimens between regions. **Table S3.** Relative abundance (%) of abundant genera. **Table S4.** Firmicutes/Bacteroidota ratio among regions, sex and age-class. **Figure S2.** Alpha diversity in Faith's PD between negative to positive fecal parasites specimens. **Table S5.** Spearman’s correlation coefficient between the quantitative measurements. **Figure S3.** PCoA plots based on dissimilarity metric demonstrated the differences between: (A) positive and negative for skin disease (Bray-Curtis) and (B) positive and negative for bone tetracycline (unweighted UniFrac). **Table S6.** Mantel correlation between quantitative measurements.

## Data Availability

16S rRNA gene reads deposited ate sequence read archive (SRA) under the bioproject number PRJEB56902.

## Abbreviations

GJ: Golden jackal
BBJ: Black-backed jackal
DD: Domestic dog
INPA: Israeli national and parks authority
