# Supplementary material for: Fecal microbiota of the synanthropic golden jackal (Canis aureus)
Source: Anim Microbiome. 2023 Aug 5;5:37. doi: 10.1186/s42523-023-00259-3 (PMC10403885; doi:10.1186/s42523-023-00259-3)
Supplement: Supplementary file 1 — Additional file 1. Figure S1. Sanitation conditions across sampling regions (1-4). Table S1. General information of GJ specimens. Table S2. Pathogen burden of GJ specimens between regions. Table S3. Relative abundance (%) of abundant genera. Table S4. Firmicutes/Bacteroidota ratio among regions, sex and age-class. Figure S2. Alpha diversity in Faith's PD between negative to positive fecal parasites specimens. Table S5. Spearman’s correlation coefficient between the quantitative measurements. Figure S3. PCoA plots based on dissimilarity metric demonstrated the differences between: (A) positive and negative for skin disease (Bray-Curtis) and (B) positive and negative for bone tetracycline (unweighted UniFrac). Table S6. Mantel correlation between quantitative measurements. [file 42523_2023_259_MOESM1_ESM.docx]

**Supplementary materials**


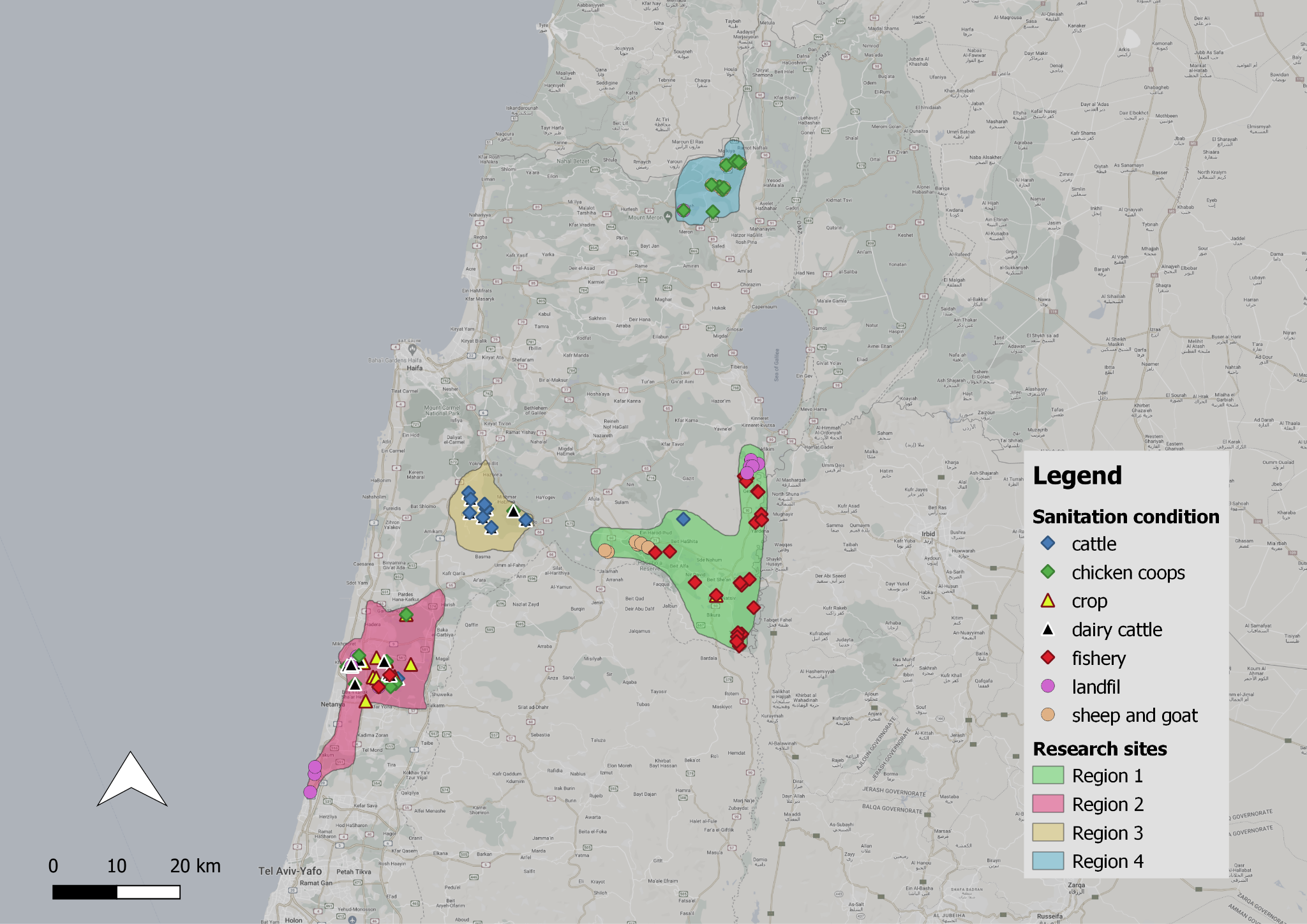


**Figure S1**. sanitation conditions across sampling regions (1-4).

**Table S1**. General information of GJ specimens

| **Animal ID** | **Geographic region** | **Sex** | **Age class** | **Weight (kg)** | **Length (cm)** | **Skin disease** | **External parasite** | **Physical condition** |
| --- | --- | --- | --- | --- | --- | --- | --- | --- |
| **R2** | **2** | **Male** | **Adult** | **8.3** | **80** | **Negative** | **Negative** | **Normal** |
| **R3** | **2** | **Male** | **Subadult** | **4.8** | **64** | **Negative** | **Negative** | **Normal** |
| **R4** | **1** | **Male** | **Subadult** | **13** | **110** | **Negative** | **Negative** | **Normal** |
| **R5** | **1** | **Female** | **Subadult** | **9.5** | **101** | **Negative** | **Negative** | **Normal** |
| **R6** | **1** | **Male** | **Juvenile** | **7.8** | **78** | **Negative** | **positive** | **Normal** |
| **R7** | **1** | **Male** | **Juvenile** | **6.5** | **70** | **Negative** | **Negative** | **Normal** |
| **R8** | **1** | **Female** | **Subadult** | **8.9** | **98** | **Negative** | **Negative** | **Normal** |
| **R9** | **1** | **Female** | **Juvenile** | **6.7** | **75** | **Negative** | **Negative** | **Normal** |
| **R10** | **1** | **Female** | **Adult** | **10** | **104** | **positive** | **Negative** | **Normal** |
| **R11** | **1** | **Male** | **Subadult** | **7.6** | **85** | **Negative** | **Negative** | **Normal** |
| **R12** | **1** | **Female** | **Juvenile** | **6.2** | **65** | **Negative** | **Negative** | **Normal** |
| **R13** | **1** | **Female** | **Juvenile** | **6.2** | **95** | **Negative** | **Negative** | **Normal** |
| **R14** | **1** | **Male** | **Juvenile** | **7.2** | **85** | **Negative** | **Negative** | **Thin** |
| **R15** | **1** | **Female** | **Subadult** | **8.4** | **103** | **Negative** | **Negative** | **Normal** |
| **R16** | **1** | **Male** | **Juvenile** | **5.5** | **89** | **Negative** | **Negative** | **Thin** |
| **R17** | **1** | **Female** | **Subadult** | **8.75** | **102** | **Negative** | **Negative** | **Normal** |
| **R18** | **1** | **Female** | **Subadult** | **8** | **102** | **Negative** | **Negative** | **Normal** |
| **R19** | **2** | **Female** | **Juvenile** | **6.85** | **95** | **Negative** | **Negative** | **Normal** |
| **R20** | **2** | **Male** | **Adult** | **13.2** | **117** | **Negative** | **Negative** | **Normal** |
| **R21** | **2** | **Female** | **Juvenile** | **5.1** | **87** | **positive** | **Negative** | **Thin** |
| **R22** | **2** | **Male** | **Subadult** | **11.3** | **105** | **Negative** | **Negative** | **Normal** |
| **R23** | **4** | **Male** | **Juvenile** | **7.5** | **103** | **Negative** | **Negative** | **Normal** |
| **R24** | **4** | **Male** | **Juvenile** | **7** | **98** | **Negative** | **Negative** | **Normal** |
| **R25** | **4** | **Male** | **Adult** | **10.5** | **103** | **Negative** | **Negative** | **Normal** |
| **R26** | **4** | **Male** | **Juvenile** | **6** | **94** | **Negative** | **Negative** | **Normal** |
| **R27** | **4** | **Female** | **Juvenile** | **5.3** | **72** | **Negative** | **Negative** | **Normal** |
| **R28** | **4** | **Male** | **Juvenile** | **8** | **96** | **Negative** | **Negative** | **Normal** |
| **R29** | **4** | **Male** | **Juvenile** | **6.3** | **103** | **Negative** | **Negative** | **Normal** |
| **R30** | **3** | **Female** | **Subadult** | **10.1** | **99** | **Negative** | **Negative** | **Normal** |
| **R31** | **3** | **Male** | **Subadult** | **9.4** | **103** | **Negative** | **Negative** | **Normal** |
| **R32** | **3** | **Male** | **Subadult** | **8.1** | **101** | **positive** | **Negative** | **Thin** |
| **R33** | **3** | **Female** | **Subadult** | **7.9** | **95** | **Negative** | **Negative** | **Thin** |
| **R34** | **3** | **Male** | **Subadult** | **10** | **95** | **Negative** | **Negative** | **Normal** |
| **R35** | **3** | **Female** | **Subadult** | **5.9** | **75** | **positive** | **Negative** | **Thin** |
| **R36** | **3** | **Male** | **Juvenile** | **6** | **83** | **positive** | **Negative** | **Normal** |
| **R37** | **2** | **Female** | **Adult** | **10.1** | **119** | **Negative** | **Negative** | **Normal** |
| **R38** | **2** | **Female** | **Adult** | **8** | **110** | **Negative** | **Negative** | **Normal** |
| **R39** | **2** | **Female** | **Adult** | **9.2** | **105** | **Negative** | **Negative** | **Normal** |
| **R40** | **2** | **Male** | **Subadult** | **10.1** | **104** | **Negative** | **Negative** | **Normal** |
| **R41** | **2** | **Female** | **Subadult** | **8.8** | **96** | **Negative** | **Negative** | **Normal** |
| **R42** | **1** | **Male** | **Subadult** | **7.8** | **103** | **Negative** | **Negative** | **Thin** |
| **R43** | **1** | **Female** | **Adult** | **10.7** | **102** | **Negative** | **Negative** | **Normal** |
| **R44** | **3** | **Female** | **Subadult** | **8.8** | **97** | **Negative** | **Negative** | **Normal** |
| **R45** | **3** | **Female** | **Adult** | **9.3** | **92** | **Negative** | **Negative** | **Normal** |
| **R46** | **1** | **Female** | **Adult** | **7.5** | **95** | **Negative** | **Negative** | **Normal** |
| **R47** | **1** | **Male** | **Adult** | **9.8** | **105** | **Negative** | **Negative** | **Normal** |
| **R48** | **1** | **Male** | **Subadult** | **9.2** | **102** | **Negative** | **positive** | **Normal** |
| **R49** | **1** | **Male** | **Adult** | **10.5** | **108** | **Negative** | **positive** | **Normal** |
| **R50** | **1** | **Female** | **Subadult** | **9.3** | **100** | **Negative** | **Negative** | **Normal** |
| **R51** | **1** | **Female** | **Adult** | **9.3** | **102** | **Negative** | **Negative** | **Normal** |
| **R52** | **2** | **Female** | **Adult** | **8.3** | **104** | **Negative** | **Negative** | **Normal** |
| **R53** | **2** | **Female** | **Subadult** | **8** | **110** | **Negative** | **Negative** | **Normal** |
| **R54** | **2** | **Female** | **Adult** | **11** | **118** | **Negative** | **Negative** | **Normal** |
| **R55** | **2** | **Male** | **Subadult** | **8.8** | **109** | **Negative** | **Negative** | **Normal** |
| **R56** | **2** | **Male** | **Adult** | **12** | **118** | **Negative** | **Negative** | **Normal** |
| **R57** | **3** | **Male** | **Subadult** | **7.8** | **90** | **Negative** | **Negative** | **Normal** |
| **R58** | **3** | **Female** | **Adult** | **8.2** | **90** | **Negative** | **Negative** | **Normal** |
| **R59** | **3** | **Male** | **Subadult** | **8.5** | **90** | **Negative** | **positive** | **Normal** |
| **R60** | **3** | **Female** | **Subadult** | **6.5** | **80** | **Negative** | **Negative** | **Normal** |
| **R61** | **3** | **Female** | **Subadult** | **5** | **80** | **Negative** | **Negative** | **Normal** |
| **R62** | **2** | **Female** | **Adult** | **9.8** | **117** | **Negative** | **Negative** | **Normal** |
| **R63** | **1** | **Female** | **Adult** | **8.5** | **103** | **Negative** | **Negative** | **Normal** |
| **R64** | **1** | **Female** | **Adult** | **8** | **99** | **Negative** | **Negative** | **Normal** |
| **R65** | **2** | **Female** | **Adult** | **11.7** | **104** | **Negative** | **Negative** | **Normal** |
| **R66** | **2** | **Male** | **Adult** | **13** | **108** | **Negative** | **Negative** | **Normal** |
| **R67** | **2** | **Female** | **Adult** | **11.5** | **102** | **Negative** | **Negative** | **Normal** |
| **R68** | **2** | **Male** | **Adult** | **11.7** | **108** | **Negative** | **Negative** | **Normal** |
| **R69** | **2** | **Male** | **Adult** | **13.2** | **124** | **Negative** | **Negative** | **Normal** |
| **R70** | **2** | **Female** | **Subadult** | **8.8** | **95** | **Negative** | **Negative** | **Normal** |
| **R71** | **2** | **Male** | **Adult** | **11.7** | **100** | **Negative** | **Negative** | **Normal** |
| **R72** | **2** | **Male** | **Adult** | **11** | **100** | **Negative** | **Negative** | **Normal** |
| **R73** | **2** | **Male** | **Adult** | **12.5** | **118** | **Negative** | **Negative** | **Normal** |
| **R74** | **2** | **Male** | **Adult** | **10** | **110** | **Negative** | **Negative** | **Normal** |
| **R75** | **2** | **Male** | **Adult** | **10.5** | **112** | **Negative** | **Negative** | **Normal** |
| **R76** | **2** | **Female** | **Adult** | **11.9** | **115** | **Negative** | **Negative** | **Normal** |
| **R77** | **2** | **Female** | **Adult** | **11.5** | **100** | **Negative** | **Negative** | **Normal** |
| **R78** | **2** | **Female** | **Adult** | **9.1** | **101** | **Negative** | **Negative** | **Normal** |
| **R79** | **2** | **Male** | **Adult** | **13.2** | **108** | **Negative** | **Negative** | **Normal** |
| **R80** | **2** | **Female** | **Adult** | **8.7** | **95** | **positive** | **Negative** | **Normal** |
| **R81** | **2** | **Male** | **Adult** | **14** | **108** | **positive** | **Negative** | **Normal** |
| **R82** | **2** | **Female** | **Adult** | **10** | **98** | **Negative** | **Negative** | **Normal** |
| **R83** | **1** | **Male** | **Adult** | **9** | **103** | **Negative** | **positive** | **Thin** |
| **R84** | **4** | **Male** | **Adult** | **11.5** | **108** | **Negative** | **Negative** | **Normal** |
| **R85** | **1** | **Male** | **Subadult** | **9.5** | **103** | **Negative** | **Negative** | **Normal** |
| **R86** | **1** | **Female** | **Adult** | **9.3** | **103** | **Negative** | **Negative** | **Normal** |
| **R87** | **1** | **Male** | **Adult** | **11.5** | **110** | **Negative** | **Negative** | **Normal** |
| **R88** | **1** | **Male** | **Adult** | **13.5** | **114** | **positive** | **Negative** | **Normal** |
| **R89** | **1** | **Female** | **Adult** | **10.5** | **108** | **Negative** | **Negative** | **Normal** |
| **R90** | **1** | **Male** | **Adult** | **11.5** | **100** | **Negative** | **Negative** | **Normal** |
| **R91** | **2** | **Male** | **Adult** | **14.7** | **120** | **Negative** | **Negative** | **Normal** |
| **R92** | **3** | **Male** | **Adult** | **11.3** | **103** | **Negative** | **Negative** | **Normal** |
| **R93** | **3** | **Female** | **Adult** | **11.3** | **107** | **Negative** | **Negative** | **Normal** |
| **R94** | **1** | **Female** | **Adult** | **9.3** | **97** | **positive** | **Negative** | **Normal** |
| **R95** | **1** | **Female** | **Adult** | **14.3** | **107** | **positive** | **Negative** | **Normal** |
| **R96** | **2** | **Female** | **Adult** | **9.8** | **109** | **Negative** | **Negative** | **Normal** |
| **R97** | **2** | **Male** | **Adult** | **12.6** | **114** | **Negative** | **Negative** | **Normal** |
| **R98** | **2** | **Female** | **Adult** | **13.3** | **110** | **Negative** | **Negative** | **Normal** |
| **R99** | **1** | **Male** | **Adult** | **11.7** | **103** | **Negative** | **Negative** | **Normal** |
| **R100** | **1** | **Male** | **Adult** | **11.2** | **102** | **Negative** | **Negative** | **Normal** |
| **R101** | **4** | **Male** | **Adult** | **10** | **102** | **Negative** | **Negative** | **Normal** |
| **R102** | **4** | **Female** | **Adult** | **12** | **102** | **positive** | **Negative** | **Normal** |
| **R103** | **4** | **Male** | **Adult** | **10** | **96** | **Negative** | **Negative** | **Normal** |
| **R104** | **4** | **Female** | **Subadult** | **10** | **96** | **Negative** | **Negative** | **Normal** |
| **R105** | **4** | **Female** | **Subadult** | **9** | **94** | **Negative** | **Negative** | **Normal** |
| **R106** | **4** | **Female** | **Adult** | **13** | **103** | **positive** | **Negative** | **Normal** |
| **R107** | **4** | **Female** | **Adult** | **10** | **105** | **Negative** | **Negative** | **Normal** |
| **R108** | **4** | **Female** | **Adult** | **12** | **105** | **Negative** | **Negative** | **Normal** |
| **R109** | **1** | **Male** | **Adult** | **13.5** | **105** | **Negative** | **Negative** | **Normal** |
| **R110** | **1** | **Male** | **Adult** | **11.8** | **102** | **Negative** | **Negative** | **Normal** |
| **R111** | **1** | **Female** | **Adult** | **12.1** | **101** | **Negative** | **Negative** | **Normal** |
| **R112** | **1** | **Female** | **Subadult** | **9.6** | **100** | **Negative** | **Negative** | **Normal** |

**Table S2**. Pathogen burden of GJ specimens between regions

| **Test type** | **Positive/Negative** | **Region** | | | | **Total** |
| --- | --- | --- | --- | --- | --- | --- |
|  |  | **1** | **2** | **3** | **4** |  |
| **Rabies detection**  **(immunofluorescent test)** | **Positive** | **0** | **0** | **0** | **0** | **0** |
|  | **Negative** | **40** | **39** | **16** | **16** | **111** |
|  | **% positive** | **0** | **0** | **0** | **0** | **0** |
| **Rabies antibodies detection** | **Positive** | **6** | **4** | **1** | **2** | **13** |
|  | **Negative** | **27** | **30** | **13** | **13** | **83** |
|  | **% positive** | **18.18** | **11.76** | **7.14** | **13.33** | **13.54** |
| **Exposure to Rabies oral vaccination (tetracycline test from bone)** | **Positive** | **26** | **17** | **4** | **9** | **56** |
|  | **Negative** | **14** | **18** | **11** | **7** | **50** |
|  | **% positive** | **65.00** | **48.57** | **26.67** | **56.25** | **52.83** |
| **Distemper virus detection**  **(brain PCR test)** | **Positive** | **5** | **1** | **0** | **3** | **9** |
|  | **Negative** | **30** | **32** | **9** | **12** | **83** |
|  | **% positive** | **14.29** | **3.03** | **0.00** | **20.00** | **9.78** |
| **Brucella serological test** | **Positive** | **0** | **0** | **0** | **0** | **0** |
|  | **Negative** | **14** | **27** | **8** | **7** | **56** |
|  | **% positive** | **0** | **0** | **0** | **0** | **0** |
| **Q-fever serological test** | **Positive** | **2** | **1** | **0** | **0** | **3** |
|  | **Negative** | **18** | **26** | **10** | **7** | **61** |
|  | **% positive** | **10.00** | **3.70** | **0.00** | **0.00** | **4.69** |
| **Leptospira serological test** | **Positive** | **1** | **0** | **0** | **0** | **1** |
|  | **Negative** | **19** | **20** | **10** | **15** | **64** |
|  | **% positive** | **5.00** | **0.00** | **0.00** | **0.00** | **1.54** |
| **Nesopora serological test** | **Positive** | **4** | **10** | **0** | **3** | **17** |
|  | **Negative** | **35** | **26** | **16** | **12** | **89** |
|  | **% positive** | **10.26** | **27.78** | **0.00** | **20.00** | **16.04** |
| **Toxoplasma serological test** | **Positive** | **11** | **12** | **5** | **3** | **31** |
|  | **Negative** | **28** | **24** | **11** | **12** | **75** |
|  | **% positive** | **28.21** | **33.33** | **31.25** | **20.00** | **29.25** |
| **Internal parasite detection (fecal)** | **Positive** | **6** | **8** | **4** | **2** | **20** |
|  | **Negative** | **5** | **8** | **0** | **4** | **17** |
|  | **% positive** | **54.55** | **50.00** | **100.00** | **33.33** | **54.05** |
| **Internal parasite detection (diaphragmatic)** | **Positive** | **4** | **3** | **1** | **2** | **10** |
|  | **Negative** | **10** | **17** | **3** | **6** | **36** |
|  | **% positive** | **28.57** | **15.00** | **25.00** | **25.00** | **21.74** |

**Table S3**. Relative abundance (%) of abundant genera

| **Genus** | **mean** | **max** | **min** | **Q1** | **Q2** | **Q3** | **Q4** |
| --- | --- | --- | --- | --- | --- | --- | --- |
| **Fusobacterium** | 23.93 | 55.73 | 0 | 17.90 | 24.61 | 32.60 | 55.73 |
| **Bacteroides** | 20.82 | 44.58 | 0 | 14.43 | 22.55 | 27.34 | 44.58 |
| **Alloprevotella** | 6.98 | 21.47 | 0 | 2.22 | 5.58 | 11.38 | 21.47 |
| **Anaerobiospirillum** | 6.53 | 28.17 | 0 | 2.45 | 4.99 | 8.63 | 28.17 |
| **Helicobacter** | 5.31 | 74.38 | 0 | 0.20 | 1.08 | 4.20 | 74.38 |
| **Prevotella** | 3.75 | 40.70 | 0 | 0 | 0 | 1.19 | 40.7 |
| **Prevotellaceae_Ga6A1_group** | 2.67 | 14.93 | 0 | 0 | 0.84 | 4.24 | 14.93 |
| **Escherichia-Shigella** | 2.23 | 44.23 | 0 | 0 | 0.49 | 1.50 | 44.23 |
| **Sutterella** | 2.10 | 6.77 | 0 | 0.95 | 2.05 | 2.97 | 6.77 |
| **Megamonas** | 1.56 | 17.27 | 0 | 0 | 0.04 | 1.33 | 17.27 |
| **Phascolarctobacterium** | 1.46 | 6.38 | 0 | 0.50 | 1.19 | 2.06 | 6.38 |
| **Faecalibacterium** | 1.21 | 8.08 | 0 | 0.07 | 0.43 | 1.54 | 8.08 |
| **f. Lachnospiraceae** | 1.03 | 3.75 | 0 | 0.25 | 0.76 | 1.54 | 3.75 |
| **Blautia** | 0.97 | 4.83 | 0 | 0.23 | 0.54 | 1.33 | 4.83 |
| **[Ruminococcus]_gnavus_group** | 0.96 | 15.15 | 0 | 0.05 | 0.26 | 1.04 | 15.15 |
| **Muribaculaceae** | 0.90 | 6.75 | 0 | 0.08 | 0.35 | 1.175 | 6.75 |
| **Peptoclostridium** | 0.76 | 5.37 | 0 | 0.28 | 0.53 | 0.93 | 5.37 |
| **Clostridium_sensu_stricto_1** | 0.73 | 9.18 | 0 | 0 | 0.07 | 0.83 | 9.18 |
| **Rikenellaceae_RC9_gut_group** | 0.72 | 9.38 | 0 | 0 | 0.08 | 0.94 | 9.38 |
| **Parabacteroides** | 0.65 | 5.48 | 0 | 0.07 | 0.32 | 0.78 | 5.48 |
| **f. Lachnospiraceae** | 0.64 | 3.40 | 0 | 0.15 | 0.42 | 0.87 | 3.4 |
| **Lactobacillus** | 0.53 | 38.87 | 0 | 0 | 0 | 0 | 38.87 |
| **Parasutterella** | 0.50 | 5.95 | 0 | 0 | 0.12 | 0.69 | 5.95 |
| **[Ruminococcus]_torques_group** | 0.47 | 6.25 | 0 | 0 | 0.18 | 0.48 | 6.25 |
| **Lachnoclostridium** | 0.44 | 4.30 | 0 | 0 | 0.28 | 0.56 | 4.3 |
| **Collinsella** | 0.42 | 8.42 | 0 | 0 | 0.13 | 0.38 | 8.42 |
| **Catenibacterium** | 0.41 | 23.65 | 0 | 0 | 0 | 0 | 23.65 |
| **UCG-005** | 0.37 | 4.15 | 0 | 0 | 0.03 | 0.41 | 4.15 |
| **Odoribacter** | 0.37 | 3.62 | 0 | 0 | 0.11 | 0.63 | 3.62 |
| **Megasphaera** | 0.37 | 12.62 | 0 | 0 | 0 | 0 | 12.62 |
| **Cetobacterium** | 0.33 | 15.50 | 0 | 0 | 0 | 0.06 | 15.5 |
| **o. Enterobacterales** | 0.32 | 12.95 | 0 | 0 | 0 | 0 | 12.95 |
| **Aeromonas** | 0.29 | 31.18 | 0 | 0 | 0 | 0 | 31.18 |
| **Succinivibrio** | 0.27 | 3.77 | 0 | 0 | 0 | 0.14 | 3.77 |
| **Anaeroplasma** | 0.27 | 4.42 | 0 | 0 | 0 | 0.16 | 4.42 |
| **Treponema** | 0.26 | 14.68 | 0 | 0 | 0 | 0 | 14.68 |
| **Campylobacter** | 0.25 | 4.27 | 0 | 0 | 0.03 | 0.20 | 4.27 |
| **Holdemanella** | 0.23 | 5.25 | 0 | 0 | 0 | 0.13 | 5.25 |
| **f. Succinivibrionaceae** | 0.22 | 8.65 | 0 | 0 | 0 | 0.01 | 8.65 |
| **Hathewaya** | 0.22 | 6.15 | 0 | 0 | 0 | 0 | 6.15 |
| **Proteus** | 0.21 | 5.90 | 0 | 0 | 0 | 0.06 | 5.90 |
| **Peptostreptococcus** | 0.21 | 6.38 | 0 | 0 | 0 | 0.08 | 6.38 |
| **Pseudomonas** | 0.20 | 6.13 | 0 | 0 | 0 | 0 | 6.13 |
| **Clostridia_UCG-014** | 0.20 | 3.35 | 0 | 0 | 0 | 0.25 | 3.35 |
| **Bifidobacterium** | 0.18 | 4.42 | 0 | 0 | 0 | 0 | 4.42 |
| **Edwardsiella** | 0.17 | 8.90 | 0 | 0 | 0 | 0 | 8.90 |
| **[Eubacterium]_coprostanoligenes_group** | 0.16 | 2.73 | 0 | 0 | 0 | 0 | 2.73 |
| **Porphyromonas** | 0.15 | 4.62 | 0 | 0 | 0 | 0 | 4.62 |
| **Paeniclostridium** | 0.15 | 2.92 | 0 | 0 | 0 | 0 | 2.92 |
| **f. Enterobacteriaceae** | 0.15 | 9.78 | 0 | 0 | 0 | 0 | 9.78 |
| **Desulfovibrio** | 0.14 | 1.83 | 0 | 0 | 0 | 0.13 | 1.83 |
| **uncultured** | 0.14 | 3.65 | 0 | 0 | 0 | 0.03 | 3.65 |
| **Clostridia_vadinBB60_group** | 0.13 | 4.65 | 0 | 0 | 0 | 0 | 4.65 |
| **[Eubacterium]_eligens_group** | 0.13 | 4.97 | 0 | 0 | 0 | 0 | 4.97 |
| **Romboutsia** | 0.13 | 4.05 | 0 | 0 | 0 | 0.01 | 4.05 |
| **Unassigned** | 0.13 | 2.28 | 0 | 0 | 0.03 | 0.13 | 2.28 |
| **Sphaerochaeta** | 0.12 | 9.33 | 0 | 0 | 0 | 0 | 9.33 |
| **Enterococcus** | 0.11 | 6.40 | 0 | 0 | 0 | 0 | 6.40 |
| **Erysipelotrichaceae_UCG-003** | 0.11 | 3.72 | 0 | 0 | 0 | 0 | 3.72 |
| **Other** | 4.64 | 256.06 | 0 | 0 | 0.00 | 0.69 | 256.06 |
| **total** | **100.00** |  |  |  |  |  |  |

**Table S4**. Firmicutes/Bacteroidota ratio among regions, sex and age-class

| **Firmicutes/Bacteroidota ratio** | | **n** | **mean (±SD)** | **min** | **max** |
| --- | --- | --- | --- | --- | --- |
| **All specimens** | | 110 | 0.69±1.41 | 0.11 | 14.6 |
| **Regional group** | **1** | 40 | 0.717±2.22 | 0.134 | 14.2 |
|  | **2** | 38 | 0.781±0.766 | 0.11 | 3.55 |
|  | **3** | 16 | 0.639±0.463 | 0.236 | 2.08 |
|  | **4** | 16 | 0.417±0.446 | 0.14 | 2.02 |
| **Sex** | **Male** | 53 | 0.797±1.95 | 0.11 | 14.2 |
|  | **Female** | 57 | 0.579±0.633 | 0.14 | 3.55 |
| **Age-class** | **Adult** | 62 | 0.587±0.676 | 0.11 | 3.55 |
|  | **Sub-adult** | 16 | 0.447±0.475 | 0.134 | 2.02 |
|  | **Juvenile** | 32 | 0.989±2.44 | 0.139 | 14.2 |


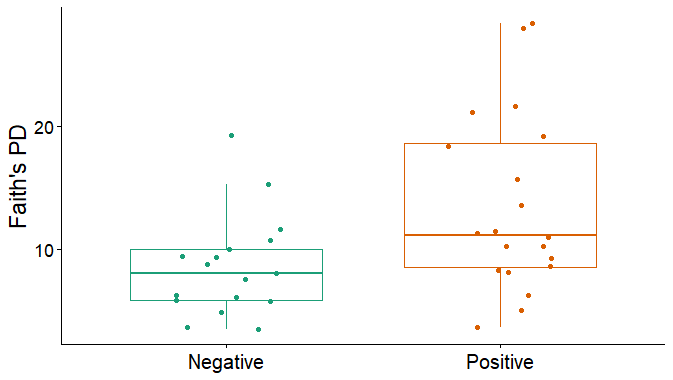


**Figure S2.** .Alpha diversity in Faith's PD between negative to positive fecal parasites specimens

**Table S5.** Spearman’s correlation coefficient between the quantitative measurements

|  | **Measure of correlation** | **Sample size** | **Test statistic** | **P-value** |
| --- | --- | --- | --- | --- |
| **weight (kg)** | Spearman | 110 | -0.0436 | 0.651 |
| **length (cm)** | Spearman | 110 | 0.0729 | 0.449 |
| **Annual precipitation (mm)** | Spearman | 110 | 0.0287 | 0.7661 |
| **Annual mean temperature** | Spearman | 110 | 0.0648 | 0.5015 |
| **Warmest month temperature** | Spearman | 110 | -0.0379 | 0.6941 |
| **Coldest month temperature** | Spearman | 110 | 0.122 | 0.2043 |

**(A)**

**
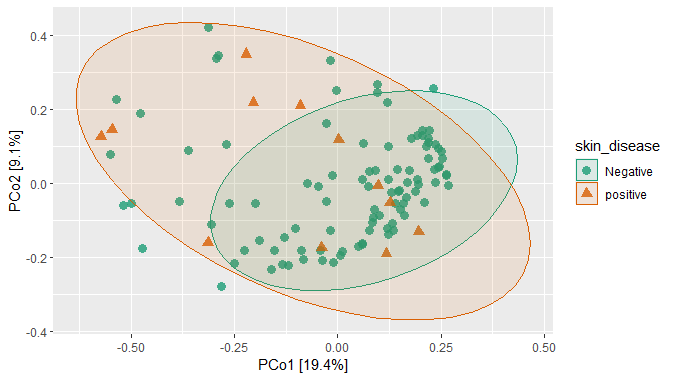
**

**(B)**
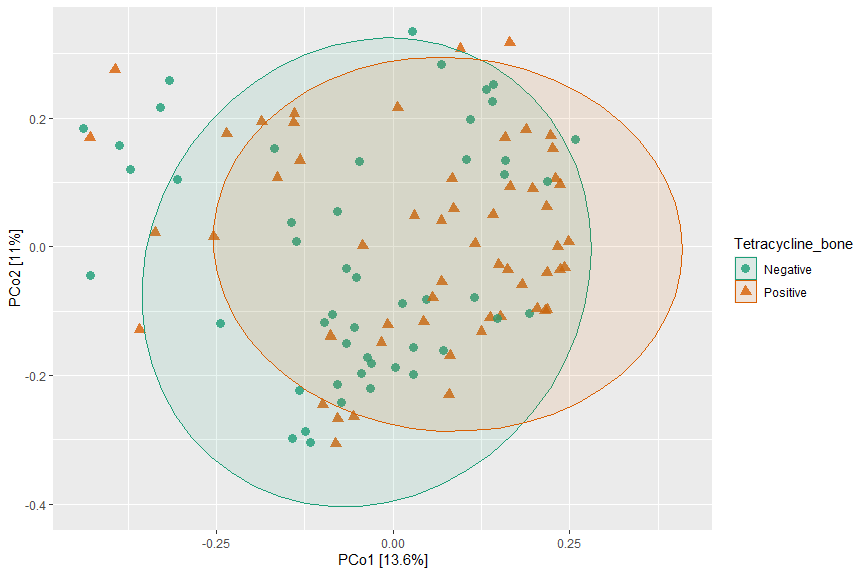


**Figure S3**. PCoA plots based on  dissimilarity metric demonstrated the differences between: (A) positive and negative for skin disease (Bray-Curtis) and (B) positive and negative for bone tetracycline (unweighted UniFrac).

**Table S6.** Mantel correlation was used to test correlation between the quantitative measurements

|  | **Measure of correlation** | **Measure of beta diversity** | **Sample size** | **Permutations** | **Alternative hypothesis** | **Spearman rho** | **p-value** |
| --- | --- | --- | --- | --- | --- | --- | --- |
| weight (kg) | Spearman | Bray-Curtis | 110 | 999 | two-sided | 0.161 | 0.003 |
| length (cm) | Spearman | Bray-Curtis | 110 | 999 | two-sided | 0.146 | 0.018 |
| Annual precipitation (mm) | Spearman | Bray-Curtis | 110 | 999 | two-sided | 0.012 | 0.584 |
| Annual mean temperature | Spearman | Bray-Curtis | 110 | 999 | two-sided | 0.005 | 0.584 |
| Warmest month temperature | Spearman | Bray-Curtis | 110 | 999 | two-sided | -0.016 | 0.604 |
| Coldest month temperature | Spearman | Bray-Curtis | 110 | 999 | two-sided | 0.0367 | 0.584 |
